# Genetic Characterization and Pathogenesis of Three Novel Reassortant H5N2 Viruses in South Korea, 2018

**DOI:** 10.3390/v13112192

**Published:** 2021-10-30

**Authors:** Anh Thi Viet Nguyen, Vui Thi Hoang, Haan Woo Sung, Seon-Ju Yeo, Hyun Park

**Affiliations:** 1 Zoonosis Research Center, Department of Infection Biology, School of Medicine, Wonkwang University, Iksan 54538, Korea; nguyenthivietanh.k56@hus.edu.vn (A.T.V.N.); hoangvui169@gmail.com (V.T.H.); 2 College of Veterinary Medicine, Kangwon National University, Chuncheon 24341, Korea; sunghw@kangwon.ac.kr; 3 Department of Tropical Medicine and Parasitology, College of Medicine, Seoul National University, Seoul 03080, Korea

**Keywords:** avian influenza virus, H5N2 low pathogenic, reassortment virus, South Korea, pathogenicity in mice

## Abstract

The outbreaks of H5N2 avian influenza viruses have occasionally caused the death of thousands of birds in poultry farms. Surveillance during the 2018 winter season in South Korea revealed three H5N2 isolates in feces samples collected from wild birds (KNU18-28: A/Wild duck/South Korea/KNU18-28/2018, KNU18-86: A/Bean Goose/South Korea/KNU18-86/2018, and KNU18-93: A/Wild duck/South Korea/KNU18-93/2018). Phylogenetic tree analysis revealed that these viruses arose from reassortment events among various virus subtypes circulating in South Korea and other countries in the East Asia–Australasian Flyway. The NS gene of the KNU18-28 and KNU18-86 isolates was closely related to that of China’s H10N3 strain, whereas the KNU18-93 strain originated from the H12N2 strain in Japan, showing two different reassortment events and different from a low pathogenic H5N3 (KNU18-91) virus which was isolated at the same day and same place with KNU18-86 and KNU18-93. These H5N2 isolates were characterized as low pathogenic avian influenza viruses. However, many amino acid changes in eight gene segments were identified to enhance polymerase activity and increase adaptation and virulence in mice and mammals. Experiments reveal that viral replication in MDCK cells was quite high after 12 hpi, showing the ability to replicate in mouse lungs. The hematoxylin and eosin-stained (H&E) lung sections indicated different degrees of pathogenicity of the three H5N2 isolates in mice compared with that of the control H1N1 strain. The continuing circulation of these H5N2 viruses may represent a potential threat to mammals and humans. Our findings highlight the need for intensive surveillance of avian influenza virus circulation in South Korea to prevent the risks posed by these reassortment viruses to animal and public health.

## 1. Introduction

Influenza viruses infect many animal species in addition to humans. Avian influenza viruses (AIVs) have a very wide host range, including mallard ducks, wild ducks, chickens, bean gooses, shorebirds, pigs, and swans. There are 16 hemagglutinin (HA) and 9 neuraminidase (NA) subtypes that were classified according to the basic surface proteins (H17-18 and N10-11 were identified in bats) [1,2]. Viruses are sub-classified into highly pathogenic avian influenza viruses (HPAIVs) and low pathogenic avian influenza viruses (LPAIVs) based on their virulence. In general, LPAIVs do not cause serious diseases in domestic birds. Most outbreaks of LPAI influenza viruses have limitations in their geographical extent and duration [3].

H5 and H7 subtypes LPAIVs evolve into HPAI viruses through a change in the HA cleavage site and cause an infectious disease in humans. The outbreak of avian influenza viruses significantly affects both the human health and the economy. The outbreak of H5N1 in Hong Kong in 1997 reported six cases of death [4]. In 1983, an outbreak of the HPAIV H5N2 in the Northeastern United States incurred a loss of approximately 65 million dollars, and Italy suffered a loss of more than 100 million dollars due to the H7N1 outbreak in 1999 [5]. The outbreak of H5N2 has been leading to the culling of millions of birds worldwide. In December 2017, a highly pathogenic H5N2 virus was detected in Russia, resulting in the culling of more than 660,000 birds in Kostroma Oblast, Central Federal District [6]. Okamatsu et al. reported the outbreak of LPAIV H5N2 in Japan during 2005–2006 [7]. In Korea, Kim et al. characterized two LPAIV isolates of H5N2 strains from 2008 to 2009 and showed that H5N2 viruses did not replicate well in chickens and mice, and the reassortment of these H5N2 viruses must have occurred in domestic ducks [8]. The Sw/Korea/C12/08 (H5N2) and Sw/Korea/C13/08 (H5N2) viruses which were isolated in pigs were reported by Lee et al. in 2009. Their experiment indicated that the Sw/Korea/C13/08 virus was more adapted and more readily transmitted than the purely avian-like virus in a swine experimental model, but not in ferrets, and the swine-adapted H5N2 viruses could be a potential model for pandemic or outbreak of HPAI [9].

Yeo et al. have reported the low-pathogenic avian influenza H5N3 reassortment virus (A/mallard duck/South Korea/KNU18-91/2018) during AIV surveillance in South Korea in the 2018-winter season. This H5N3 virus has resulted from the reassortment event of multiple virus strains coming from China and Mongolia via migratory birds [10]. In this study, we analyzed the genetic and molecular characterizations of three novel H5N2 isolates at the same time surveillance in 2018 (one strain was isolated from bean goose, the others were isolated from wild duck) to obtain more knowledge regarding the circulation of H5 AIVs in South Korea. Additionally, we investigated the pathogenesis of H5N2 AIVs in mammalian cell and animal models (in vitro and in vivo).

## 2. Materials and Methods

### 2.1. Collection of Feces Sample

Fresh wild bird’s feces samples were collected in South Korea environment by sterile swabs in the 2018-winter season. The feces samples were maintained at 2–8 °C and then doing further analysis within 12 h in the laboratory.

### 2.2. Virus Isolation from the Feces Sample

Phosphate buffered saline (PBS; pH 7.4) containing 100 U/mL penicillin and 100 mg/mL streptomycin was used to resuspend the feces samples. The mixed samples were centrifuged 3000 rpm at 4 °C for 10 min to collect the supernatant, which was then filtered by a 0.45 µm pore size GVS Syringe Filter (Nova-Tech, Kingwood, TX, USA). Next, virus samples were inoculated in 10-day-old specific-pathogen-free (SPF) embryonated chicken eggs (ECEs) (Seng-Jin Inc., Eumsung, Korea) for virus amplification at 37 °C for 72 h under humidified conditions. Allantoic fluid was collected from the inoculated ECEs, and the hemagglutination assay was performed to verify the presence of the influenza virus.

### 2.3. RNA Extraction for Sequencing

Viral RNA was extracted directly from the allantoic fluid of ECEs using the NucleoSpin^®^ RNA extraction kit (Macherey-Nagel, Düren, Germany) according to the manufacturer’s instructions. RNA was finally eluted in 60 µL of RNase-free water, supplied with 20 units of RNase inhibitor for storage at −80 °C for further analysis. A reverse transcription polymerase chain reaction (RT-PCR) was performed to verify the presence of the influenza virus in the sample. The matrix gene was amplified using universal primers and probes according to the World Health Organization guidelines [11]. Host identification was confirmed by DNA barcoding using primers for identifying the mitochondrial cytochrome oxidase I (COI) gene in DNA isolated from feces samples, as described previously [12].

### 2.4. Next Generation Sequencing (NGS) by Illumina Hiseq X Method

Next generation sequencing was conducted by GnCBio (Daejeon, South Korea) on the Illumina Hiseq X platform (Illumina Inc., San Diego, CA, USA), as previously reported [13]. Briefly, viral RNA quality was determined using an Agilent RNA 6000 Pico kit (Agilent Technologies Inc., Santa Clara, CA, USA), and the concentration was measured using a spectrophotometer. The cDNA library of viral RNA was determined using a QIAseq FX single cell RNA library kit (QIAGEN, Hilden, Germany). Library concentration was measured using a LightCycler qPCR (Roche, Basel, Switzerland) and library size was checked using the TapeStation HS D5000 screen tape (Agilent Technologies Inc.). For cluster generation, the library was loaded into a flow cell where fragments were captured on a lawn of surface-bound oligos complementary to the library adapters. Each fragment was subsequently amplified into distinct clonal clusters through bridge amplification. When cluster generation was complete, the templates were used for sequencing. Sequencing data were converted into raw data for analysis.

### 2.5. Phylogenetic Tree Analysis

Nucleotide blast analysis was used to identify the closest relatives of the viral genes. All reference sequences were downloaded from Global Initiative on Sharing All Influenza Data (GISAID, http://www.gisaid.org, accessed on 1 September 2021) and the National Center for Biotechnology Information (NCBI, https://www.ncbi.nlm.nih.gov, accessed on 1 September 2021). The blasted sequence of all three isolates was combined, and the replicated sequences were deleted. Next, phylogenetic trees were generated by the maximum likelihood method with 1000 bootstrap replicates using MEGA-X software version 10.2.6 (Molecular Evolutionary Genetics Analysis, Pennsylvania State University, State College, PA, USA).

### 2.6. Viral Replication Kinetics in MDCK Cells

Three isolated H5N2 viruses (A/Wild duck/South Korea/KNU18-28/2018, A/Bean Goose/South Korea/KNU18-86/2018, and A/Wild duck/South Korea/KNU18-93/2018) together with two reference strains (human-origin H1N1 (A/California/04/2009) and H5N3 (A/spot-billed duck/Korea/KNU SYG06/2006)) were infected into the Madin–Darby canine kidney (MDCK) cell line (American Type Culture Collection) to examine the viral replication capacity in mammalian cells. Briefly, five kinds of viruses were inoculated with 0.01 multiplicity of infection (MOI) into MDCK cell monolayers in DMEM containing 1 µg/mL TPCK-treated trypsin (Sigma-Aldrich, Saint Louis, MO, USA) and were incubated at 37 °C. The virus supernatant was collected every 12 h until 72 h post-infection (hpi). Viral replication titers were determined by the TCID_50_ assay combined with the enzyme-linked immunosorbent assay (ELISA) for detecting the viral nucleoprotein (NP) antigen.

### 2.7. Determination of Infectious Doses–EID_50_ and TCID_50_

The enzyme-linked immunosorbent assay (ELISA) was used to measure the 50% tissue culture infectious dose (TCID_50_) titers as reported previously [14]. Briefly, MDCK cells were grown on flat-bottom 96-well plates at 37 °C in an atmosphere containing 5% CO_2_. When MDCK cells reached 80–90% confluence, cells were washed with PBS. The cells were then inoculated with 10-fold serial dilutions of viral suspensions. The medium was removed; the cells were washed with PBS once and fixed with 80% cold acetone after 3 days of cell incubation. The plates were blocked with 5% skim milk to remove the non-specific binding for 2 h at 37 °C. Next, the anti-influenza nucleoprotein antibody (0.1 µg/well) was applied to capture the nucleoprotein fixed on the well bottom surface for 1 h at 37 °C. After washing 3 times with PBS-T, the rabbit polyclonal antibody to mouse IgG (HRP) (Abcam, Cambridge, UK) was added at 1:5000 dilution. The TMB substrate was applied after stringent washing with PBS-T five times and the signal was measured at wavelength 450 nm with a SpectraMax^®^ iD3 microplate reader (Molecular Devices, San Jose, CA, USA). The TCID_50_ titers were determined using the Reed and Muench method [15].

To measure the 50% egg infective dose (EID50), the chorioallantoic cavities of 10-day-old SPF ECEs were inoculated with 100 μL serial 10-fold dilutions of the viruses, using five ECEs for each dilution. The ECEs were incubated at 37 °C for 3 days, after which the allantoic fluid was harvested and tested for hemagglutination activity. Calculation of EID_50_ for each viral suspension was performed according to the Reed and Muench’s calculation method.

### 2.8. Adaptation in Mice

The pathogenic potential of the new isolate was determined in 6-week-old female BALB/c mice purchased from Orient (Seongnam, Gyeonggi, South Korea) (*n* = 12) that were intranasally inoculated with 10^5^ EID_50_/50 µL of the virus. Mice were anesthetized using 1% isoflurane following the manufacturer’s instructions (Hana Pharmacy, Hwasung, South Korea) and the guidelines of Vertebrate Animal Research, University of Iowa. The body weight and survival rate of mice were observed for 14 days. Mice were euthanized and their lungs were collected on days 3, 6, and 14 post-infection (*n* = 3). The lung tissue was homogenized, and the TCID_50_ was determined to test the viral titers of homogenate supernatant. This study was conducted following the relevant guidelines and regulations of the Animal Ethics Committee of the Wonkwang University (WKU19-64, 25 November 2019). The lungs from three mice collected at day 6 post-infection for each group were fixed in 10% formalin/saline, and the tissue was embedded in paraffin. By using standard hematoxylin and eosin (H&E) staining, histological characterization of the lung sections was observed under microscopy at 40× magnification.

### 2.9. Statistical Analysis

All results were statistically analyzed using GraphPad Prism 5.0. One-way and two-way analyses of variance (ANOVA) were used to analyze the bodyweight change, the lung weight and the TCID_50_ data. A value of *p* < 0.05 was considered significant. In general, all data were represented as mean and standard deviations (SD) of biological replicates.

## 3. Results

### 3.1. Genetic Characterization of Three Novel Avian Influenza A (H5N2) Viruses

Among the 30 positive samples out of the collected 500 fecal samples from various wild birds, such as mallard duck, big goose, spot-billed duck, rump pigeon, and bean goose, in 2018, three isolated viruses were determined as H5 subtype AIVs based on HA and NA gene sequencing results from NGS. The A/Wild duck/South Korea/KNU18-28/2018 (H5N2) (KNU18-28) strain was collected from Han River Estuary, Paju, Gyeonggi-do (37°45′28.79″, 126°41′42.19″) on 16 October 2018. The A/Bean Goose/South Korea/KNU18-86/2018 (H5N2) (KNU18-86) and A/Wild duck/South Korea/KNU18-93/2018 (H5N2) (KNU18-93) strains were collected from Hwaseong Sihwa Lake, Gyeonggi-do (37°12′27.49″, 126°40′3.68″) on 2 December 2018. The viruses were inoculated in ECEs and identified using hemagglutination assay. The full genome sequences of the three H5N2 isolates were submitted to the NCBI GenBank database, with accession numbers MT477766-MT477773, MT477790-MT477797, and MT477798-MT477805 corresponding to eight gene segments of KNU18-28, KNU18-86, and KNU18-96, respectively.

The highest nucleotide identities for the three isolates obtained from the GenBank database are shown in Table 1, Table 2 and Table 3. Surface genes (HA and NA) and two internal genes (NP and M) of all three isolates were closely related to isolated strains in Korea sharing 98.30% to 99.80% sequence identities, whereas remaining genes (PB2, PB1, PA, and NS) were related to isolates from other countries. PB1 and PA gene segments were most closely related to those of isolated strains from China. PB2 gene segment was related to those of the Bangladesh strain. Different from all other gene segments, internal gene NS of KNU18-28 and KNU18-86 strains was closely related to the A/Environment/Guangxi/13425/2018 (A/H10N3) strain with 99.52% and 99.64% identities, whereas only the KNU18-93 NS strain was closely related to the strain isolated in Japan (A/duck/Hokkaido/56/2017 (A/H12N2), sharing the identity of 99.76%.

**Figure 1:**
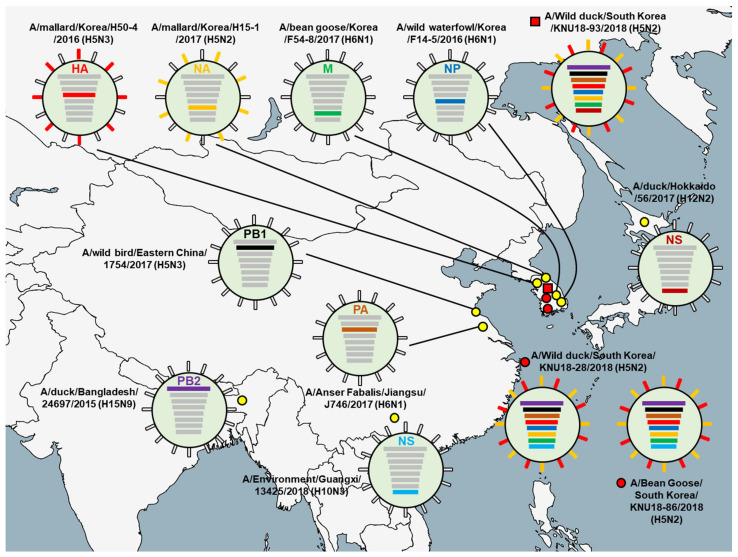
Putative origins of the genes comprising the KNU18-28 (A/Wild duck/South Korea/KNU18-28/2018 (H5N2)), KNU18-86 (A/Bean Goose/South Korea/KNU18-86/2018 (H5N2)), and KNU18-93 (A/Wild duck/South Korea/KNU18-93/2018 (H5N2)) strains.

The putative original gene segments of the KNU18-28, KNU18-86, and KNU18-93 isolates are illustrated in Figure 1. Various isolated strains circulated in Korea, Japan, China, and Bangladesh belong to the East Asian–Australasian Flyway (EAAF), indicating viral reassortment resulting in three novel H5N2 isolated strains in this study. However, there are two different reassortments of the H5N2 subtype virus at only the NS gene segment.

**Figure 2:**
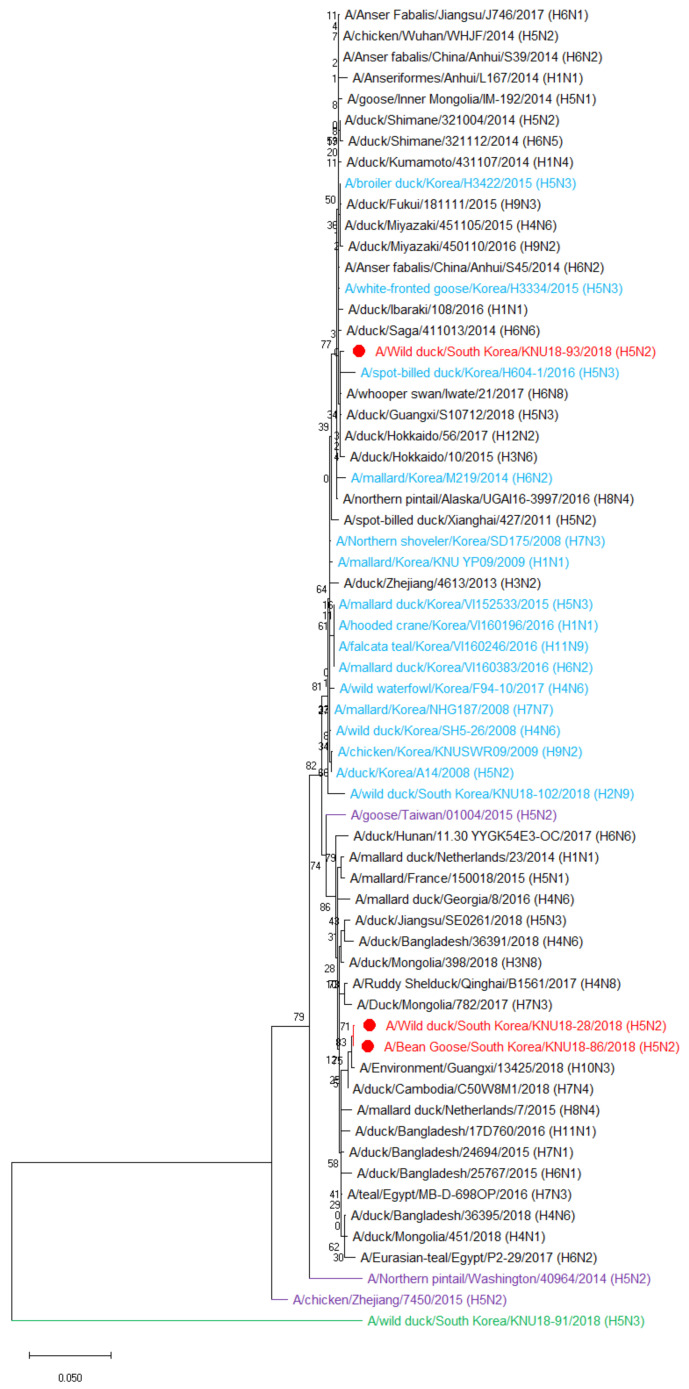
Phylogenetic tree analysis of NS gene segment based on the nucleotide sequences. MEGA-X software using maximum likelihood method with bootstrap replication (1000 bootstraps) was used to generate the phylogenetic tree. Red color: novel H5N2 isolates, blue color: Korean strains, purple color: H5N2 highly pathogenic avian influenza viruses (HPAIVs), and green color: H5N3 strain isolated at the same surveillance time.

Phylogenetic tree analysis was performed for eight genes of the three novel H5N2 isolates to evaluate their genetic relationship with those of wild birds and domestic poultry in Korea and neighboring countries. All eight genes (PB2, PB1, PA, HA, NP, NA, M, and NS) in our three selected H5N2 strains were mainly distributed in East Asian and Eurasian lineages, far from highly pathogenic H5N2 strains as well as the H5N3 isolate (KNU18-91) (Figure 2 and Figure S1). The three H5N2 isolates were similar and distributed in the same cluster for all gene segments except NS. Based on the phylogenetic tree analysis, the closest donor strains to three novel H5N2 isolates were Bangladesh H15N9 strain for PB2, Eastern China H5N3 strain for PB1, Korea H5N3 strain for HA, Korea H5N2 strain for NA, and Korea H6N1 strain for NP and M gene segments, indicating the consistent with homology identification in Table 1, Table 2 and Table 3. The PA gene was most closely related to the Korean H5N2 strain in 2017, although it showed the highest similarity with the Jiangsu H6N1 strain. As shown in Figure 2, KNU18-28 and KNU18-86 isolates were classified in one cluster, including one highly pathogenic H5N2 strain from Taiwan and close to Hokkaido H12N2 strain, whereas the KNU18-93 isolate belonged to another cluster that includes strains from Korea and one H8N4 strain from Alaska, showing the most closely related to Guangxi H10N3 strain.

### 3.2. Hypothesis of the Reassortment Event of the Three Novel H5N2 Isolated Strains (KNU18-28, KNU18-86, and KNU18-93)

**Figure 3:**
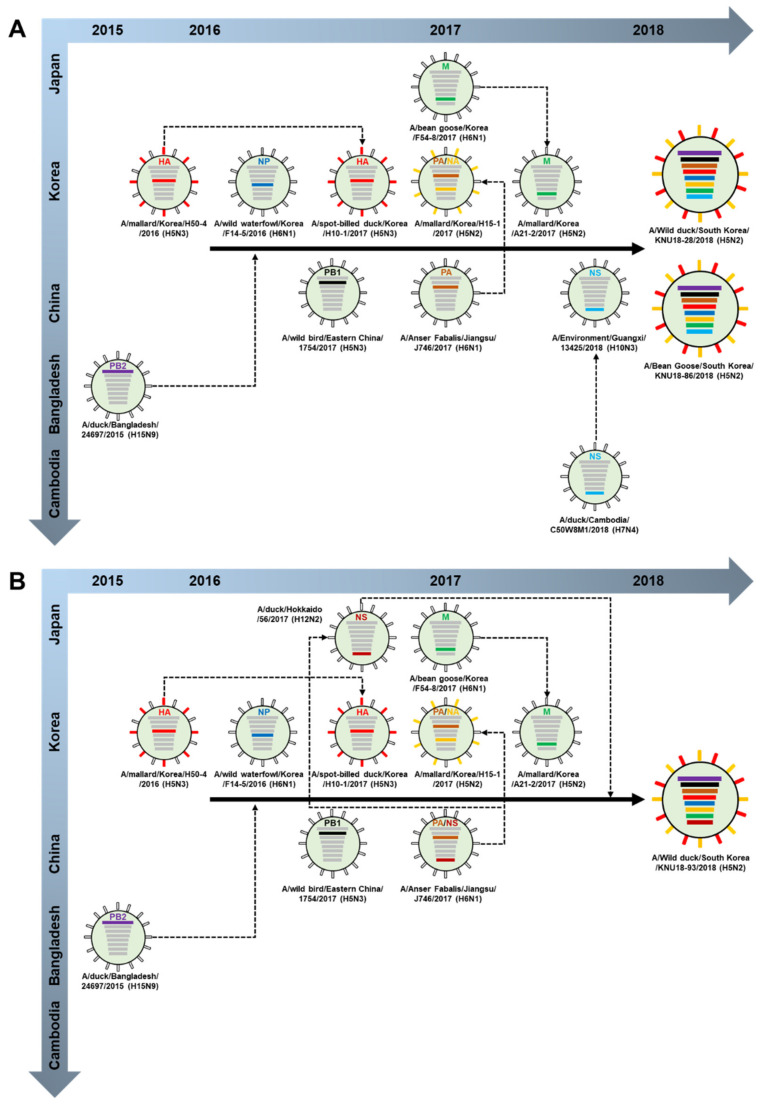
Hypothesis of the ancestor of each gene segment evolution of (**A**) KNU18-28 (A/Wild duck/South Korea/KNU18-28/2018 (H5N2)), KNU18-86 (A/Bean Goose/South Korea/KNU18-86/2018 (H5N2)), and (**B**) KNU18-93 (A/Wild duck/South Korea/KNU18-93/2018 (H5N2)) strains.

We proposed a hypothesis identifying the ancestor of each gene segment of the three H5N2 isolates in two different reassortment events as shown in Figure 3.

The ancestors of 5/8 gene segments (HA, NA and PA, NP, and M) were identified in various strains (H5N3, H5N2, H6N1, and H5N2, respectively) in Korea during the migratory season 2016–2017. A/mallard/Korea/H50-4/2016 (H5N3) may donate HA gene to the A/spot-billed duck/Korea/H10-1/2017 (H5N3), and A/bean goose/Korea/F54-8/2017 (H6N1) may donate M gene to the A/mallard/Korea/A21-2/2017 (H5N2) before the reassortment resulting in KNU18-28 and KNU18-86 strains (Figure 3A). This implies that the circulation and reassortment of migratory birds occurred in Korea during 2016–2018. In 2017, wild birds coming from China donated PB1 and PA gene segments for these reassortments. A/duck/Bangladesh/24697/2015 (H15N9) circulated in EAAF and donated PB2 gene for KNU18-28 and KNU18-86 strains in the 2018 bird migratory season. A/Environment/Guangxi/13425/2018 (H10N3) may have been reassorted with NS gene from the donor A/duck/Cambodia/C50W8M1/2018 (H7N4) before moving to Korea in late 2018.

Figure 3B presents the hypothesis of the KNU18-93 strain showing that the reassortment of all gene segments was similar to those of KNU18-28 and KNU18-86 strains, except the NS gene. A/Anser Fabalis/Jiangsu/J746/2017 (H6N1) donated the NS gene to A/duck/Hokkaido/56/2017 (H12N2); during bird migratory season 2017–2018, it migrated from Japan to Korea and was involved in the reassortment resulting in the KNU18-93 isolated strain.

### 3.3. Molecular Characterization

Table 4 represents the adaptive amino acid mutation in the HA protein of the three H5N2 isolates selected in this study (D/E94N, I116M, S121N, A134V, G139R, S142G, D221G/N, and Q222L; H5 numbering) that prefer the virus binding to α-2,6-linked sialic acid resulting in viral fusion enhancement [16,17,18,19,20]. The HA and NA genes of these isolates were compared with a low pathogenic H5N3 virus isolated in Korea in late 2018 (A/mallard duck/South Korea/KNU18-91/2018) [10], a low pathogenic H5N2 virus isolated in Korea in 2008 (A/duck/Korea/A14/2008) [8] and a highly pathogenic H5N2 strain from China (A/chicken/Zhejiang/7450/2015) [21]. The HA cleavage sites of these H5N2 isolates were characterized as monobasic residue PQRETR↓GLF, indicative of the LPAIVs. However, the NA of all three H5N2 isolates contained isoleucine at positions 26 and 223, and the HA contained glycine at position 221, which suggests increasing virulence in mammals [22,23,24,25].

While lacking key mutations that support the virus become highly pathogenic; many amino acid mutation sites of internal genes, involved in enhancing the virulence of virus in mammalian cells, birds, and mice, of all three H5N2 isolates are shown in Table 5. The three novel H5N2 isolates selected in this study have the same mutation sites in all gene segments. The gene segments (PB2, PB1, and PA) contain many mutations that facilitate polymerase activity and increase mammals adaptation and virulence in mice. The other gene segments (HA, NA, NP, M, and NS) carried mutations that were supposed to increase virulence in mice and mammals.

### 3.4. Viral Replication Kinetics in MDCK Cells

The viral replication kinetics of three H5N2 isolates in mammalian cells was investigated compared to that of the H1N1 (A/California/04/2009) and H5N3 (A/spot-billed duck/Korea/KNU SYG06/2006) control virus strains.

**Figure 4:**
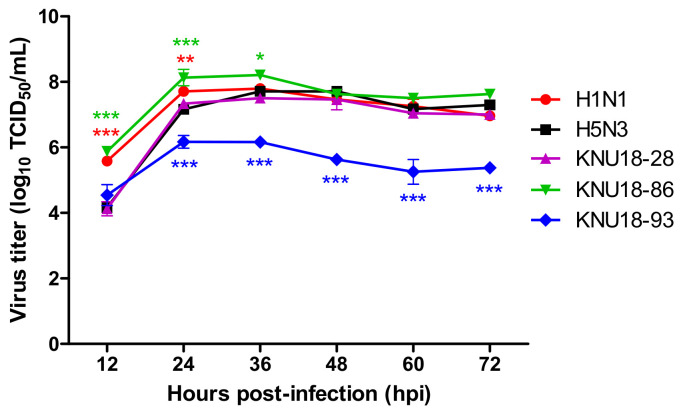
Growth kinetics replication of three novel H5N2 isolates in MDCK cells. Five kinds of viruses were infected with 0.01 multiplicity of infection (MOI) into MDCK monolayers. The virus supernatants were collected at different time points each 12 h until 71 h post-infection (hpi). The viral replication titers were then determined by the TCID_50_ assay combined ELISA for detecting the nucleoprotein (NP) viral antigen. *, *p* < 0.05; **, *p* < 0.01; and ***, *p* < 0.001 showing comparison with H5N3 control.

Figure 4 shows that all three isolated viruses reached a peak at 36 hpi; among them, the titer of the KNU18-86 strain was the highest with a curve pattern similar to that of the control H1N1 strain. The same kinetic curve pattern was observed for the KNU18-28 strain and the control H5N3 strain. In contrast, the KNU18-93 strain has a different growth curve with 20–100 folds lower titer compared with other isolates and the control virus strain. The TCID_50_ assay raw data for growth kinetics are presented in Figure S2.

### 3.5. Adaptation in Mice

**Figure 5:**
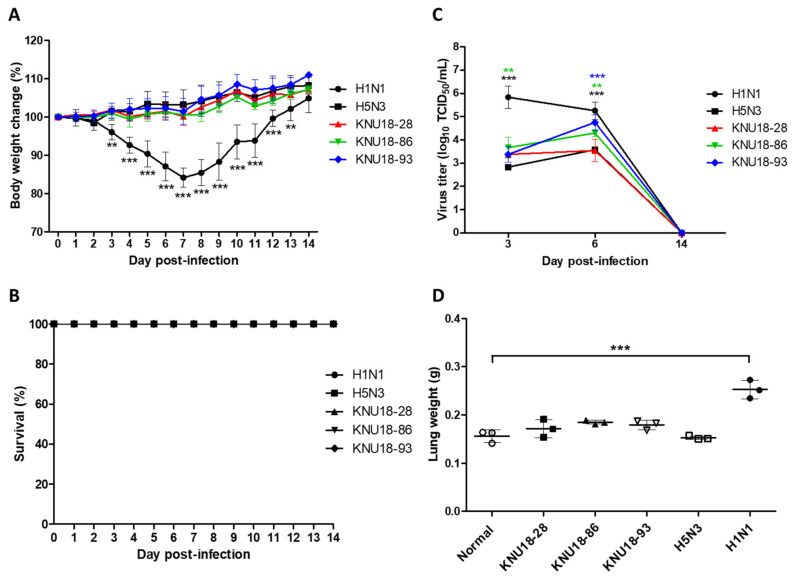
In vivo pathogenicity of three novel H5N2 isolates in mouse model. The BALB/c mice were intranasally challenged with three novel H5N2 isolates (KNU18-28, KNU18-86, and KNU18-93) at titer 10^5^ EID_50_/mouse. H1N1 and H5N3 virus strains were used as controls. Mouse bodyweight change (**A**) and survival rate (**B**) were observed for 14 days post-infection (dpi). Bodyweight is presented as a % change from those of the same mice at day 0 (*n* = 5). (**C**) The viral titer mean values in the mouse lung (*n* = 3) were determined at 3, 6, and 14 dpi. (**D**) Mean lung weight of normal mice and infected mice at 6 dpi (*n* = 3) was observed before examining the histology of lung inflammation by hematoxylin and eosin (H&E) staining. **, *p* < 0.01; ***, *p* < 0.001.

To determine the pathogenic potential in mammals, the six-week-old female BALB/c mice were used to inoculate the three novel H5N2 isolates with a 10^5^ EID_50_/50 µL. The control H1N1 and H5N3 strains were included for comparison. The body weight of the mice infected with the H1N1 strain was found to decrease regularly, with the lowest weight (84.14% ± 2.29%) observed at 7 days post-infection (dpi). In contrast, the three novel H5N2 isolates and H5N3 control strain maintained a stable body weight for 14 days (Figure 5A). All infected mice survived, showing no difference in the survival percentage between five groups (Figure 5B). The viral replication at day 3, 6, and 14 post-infection in the lungs are shown in Figure 5C. Overall, all of the virus replicated in the lung with high titer at 3 and 6 dpi and were absent in the lung at 14 dpi. The H1N1 control group showed the highest titer virus replicated in the lung (5.84 ± 0.39 log_10_ TCID_50_/mL at 3 dpi and 5.25 ± 0.31 log_10_ TCID_50_/mL at 6 dpi), whereas H5N3 control strain in the lung with the lowest titer (2.84 ± 0.21 log_10_ TCID_50_/mL at 3 dpi and 3.58 ± 0.12 log_10_ TCID_50_/mL at 6 dpi). The three novel H5N2 isolates (KNU18-28, KNU18-86, and KNU18-93) were observed with high viral replication titer at 3 dpi (3.38 ± 0.27, 3.67 ± 0.36, 3.38 ± 0.27 log_10_ TCID_50_/mL, respectively) and at 6 dpi (3.54 ± 0.39, 4.30 ± 0.12, 4.75 ± 0.27 log_10_ TCID_50_/mL, respectively). Among them, the KNU18-93 strain replicated with a low titer at 3 dpi, which then became higher than that of the KNU18-86 strain at 6 dpi. Raw data from the TCID_50_ assay are shown in Figure S3. The viral replication in the lung at 6 dpi showed a significant difference between KNU18-86 (*p* < 0.01) and KNU18-93 (*p* < 0.001) strains, compared with control strain H5N3.

**Figure 6:**
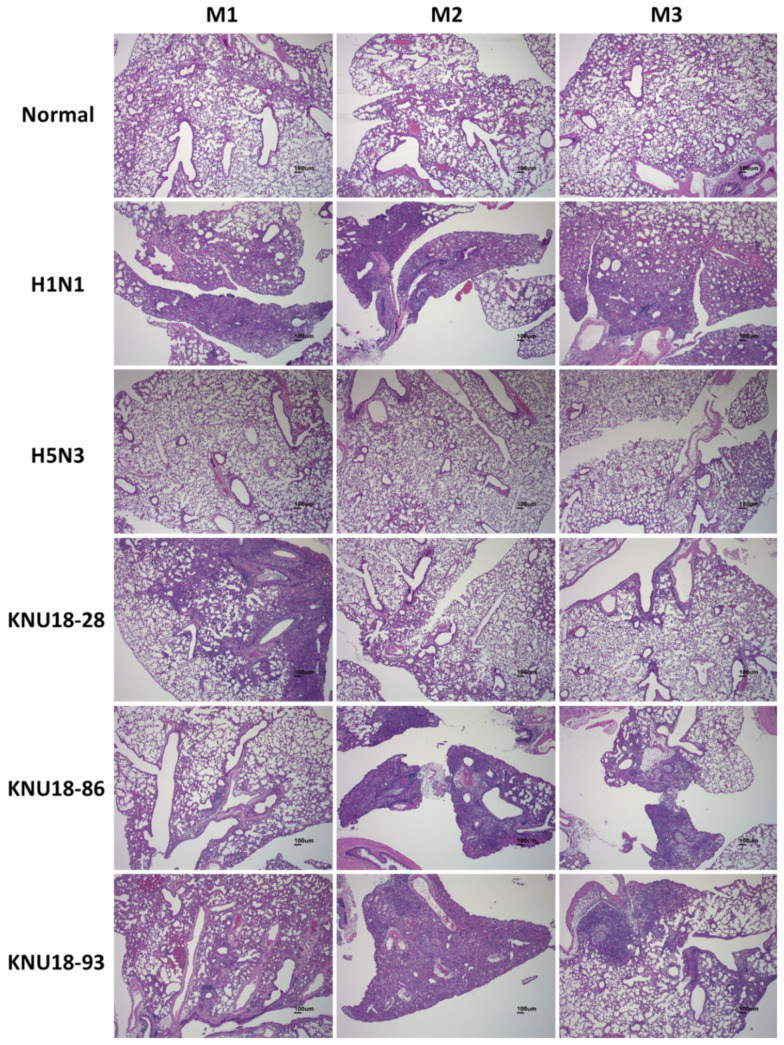
Histology of lung inflammation examined by hematoxylin and eosin (H&E) staining. BALB/c mice were intranasally challenged with 10^5^ EID_50_/mouse (*n* = 3) of three novel H5N2 isolates (KNU18-28, KNU18-86, and KNU18-93) and two control virus strains (H1N1 and H5N3). The infected mouse lungs were collected for H&E staining at 6 dpi (scale bar, 100 µm; original magnification, 40×).

Therefore, we conducted histopathological examinations of the mice lungs to compare all mice groups (*n* = 3 in each group). Except for the normal and H5N3 mice groups, the H&E-stained sections of the KNU18-28, KNU18-86, KNU18-93, and H1N1-infected lungs revealed that neutrophils had penetrated the alveolar air spaces at 6 dpi (Figure 6). All three H1N1-infected mice showed severe lung injury with a significant increase in weight than the other mice groups (Figure 5D). None of the mice infected with KNU18-28, KNU18-86, and KNU18-93 strains show any difference in lung weight; however, they showed viral replication in the lung with different patterns of pathogenicity. The real lung morphology is shown in Figure S4.

## 4. Discussion

H5N2 viruses mainly infect migratory and domestic poultry birds but not humans. Outbreaks of highly pathogenic H5N2 viruses continuously cause the death of thousands of birds in poultry farms.

Recently, various novel H5 HPAIVs have been frequently isolated from poultry and wild birds in Asia, Europe, and North America. Highly pathogenic H5N2 viruses have been characterized by the reassortment of LPAI H9N2 viruses and HPAI H5N8 viruses, clade 2.3.4.4b circulating in Egypt [50,51], British Columbia [52], and Alaska [53]. In China, HPAI H5N2 viruses emerged from HPAI H5N1 clade 2.3.4 and LPAI H9N2 or from viruses of various subtypes from the natural gene pool [54,55,56]. In this study, three novel H5N2 isolates received their genes from various virus strains, including H5N2, H5N3, H6N1, H15N9, H10N3, and H12N2, through migratory bird circulation in the EAAF. According to the phylogenetic tree, 6/8 genes of these three H5N2 AIVs were donated from Korean strains. The phylogenetic tree also reveals two different reassortment events in the NS gene segment, which may be a critical factor responsible for viral replication as reported previously [57]. All gene segments of KNU18-28 and KNU18-86 were donated from the same virus strains in the reassortment event. However, they were isolated from different hosts at different places and at different times. KNU18-86 had higher virus replication kinetics in MDCK cells and was slightly more pathogenic than KNU18-28 in experiments. In contrast, although KNU18-93 was isolated on the same day and the same place with KNU18-86, we supposed that KNU18-93 belonged to another reassortment event for the NS gene segment. The viral replication kinetic of KNU18-93 was significantly lower but showing the most pathogenic in mice at 6 dpi compared with KNU18-28 and KNU18-86. In addition, three novel H5N2 isolates share ≤98.68% sequence homology, especially <75% for NS gene with KNU18-91 (H5N3) [10] which were isolated on the same day as well as a place with KNU18-86 and KNU18-93 (Table S1). The phylogenetic trees also reveal the far distance of these H5N2 isolates with the KNU18-91 (H5N3) isolate (Figure 2 and Figure S1).

In the previous report, animal data showed that the KNU18-91 (H5N3) isolate did not replicate well in mice, showing a similar pattern and significantly lower virus titer in the lung at 3 dpi compared with the H5N3 control strain (A/spot-billed duck/Korea/KNU SYG06/2006) [10]. In our present study, we also used H5N3 (A/spot-billed duck/Korea/KNU SYG06/2006) as control and challenged mice with higher doses using 10^5^ EID_50_/mL. Compared with H5N3, we obtained a similar virus titer in mice lung infected KNU18-28 and higher virus titer in mice lung infected KNU18-86 (*p* < 0.01) and KNU18-93 (*p* < 0.001) at 6 dpi (Figure 5C). Taken together, these results suggest that numerous different H5 AIVs were reassorted and circulated in South Korea at the same time.

Apart from the cleavage site, the mutation sites also support the adaptation of avian influenza viruses to mammals, especially the E627K and D701N substitutions in the polymerase basic protein 2 (PB2). Both the key mutations, E627K plays a critical role in mammalian adaptation [58,59] and D701N facilitates viral polymerase activity in mammals [60,61], were not detected in these three novel H5N2 isolates. However, six other mutations in the PB2 gene were all detected in three novel H5N2 isolates, including L89V, G309D, T339K, R447G, I495V, and A676T. These multiple mutation sites can compensate for the E627K mutation effect in mice [26], suggesting the increase in virulence of three novel H5N2 isolates in mice.

Host adaptation for virulence is generally performed through 5 to 20 lung-to-lung passages in mouse models. In 2017, Nam et al. reported that a low pathogenic avian influenza H5N2 virus, A/Aquatic Bird/Korea/CN2/2009, shifted rapid virulence during a single passage in mice lungs. All mice infected with the passage 1 virus died within 8 dpi, indicating the potential of the virus to become virulent with 100% lethality after a single passage in mice. The molecular analysis indicated PB2 E627K and PA T97I mutations that are responsible for the acquisition of mouse virulence in a common LPAI H5N2 isolate after a single passage [62]. In this study, although these H5N2 strains were identified as LPAI, they were found to be pathogenic in mice with the ability to replicate in mice lungs. No significant difference was observed in body- and lung-weight changes for all infected mice groups except H1N1. However, 1/3 KNU18-28-infected mice, 2/3 KNU18-86-infected mice, and 3/3 KNU18-93-infected mice showed moderate to severe lung injury, compared with H1N1-infected mice (3/3) in Figure 6. Our result revealed that these three LPAI H5N2 viruses have the potential to adapt and become more virulent in mice and mammals.

Our present study indicates that H5 AIVs circulating in Korea have undergone reassortment with other AIVs in Korea, as well as in other countries, via bird migration, resulting in two genetic groups of novel reassorted H5N2 AIVs causing virulence in mice. Thus, continuously monitoring AIVs in wild migratory birds has become a critical task in the prevention and control of the AIV in South Korea.

## Figures and Tables

**Table 1:** Sequence homology of the whole A/Wild duck/South Korea/KNU18-28/2018 (A/H5N2) genome.

Gene Segment	Genbank ID	Reference Strain Accession ID	Highest Similarly Reference Strain	Identity (%)
PB2	MT477766.1	EPI965204 EPI965285 EPI1332121	A/duck/Bangladesh/24697/2015 (A/H15N9) A/duck/Bangladesh/24704/2015 (A/H15N9) A/Environment/Jiangxi/22207/2014 (A/H4N2)	99.12 (2260/2280) 99.08 (2259/2280) 98.86 (2254/2280)
PB1	MT477767.1	EPI1549213 EPI1549229 EPI1549221	A/wild bird/Eastern China/1754/2017 (A/H5N3) A/wild bird/Eastern China/1759/2017 (A/H5N3) A/wild bird/Eastern China/1758/2017 (A/H5N3)	99.34 (2259/2274) 99.30 (2258/2274) 99.30 (2258/2274)
PA	MT477768.1	EPI1619612 EPI1848485 EPI1513958	A/Anser Fabalis/Jiangsu/J746/2017 (A/H6N1) A/Environment/Guangxi/13425/2018 (A/H10N3) A/mallard/Korea/H15-1/2017 (A/H5N2)	99.44 (2139/2151) 99.40 (2138/2151) 99.40 (2138/2151)
HA	MT477769.1	EPI1513834 EPI1528984 EPI1514074	A/mallard/Korea/H50-4/2016 (A/H5N3) A/duck/Jiangsu/SE0261/2018 (A/H5N3) A/spot-billed duck/Korea/H10-1/2017 (A/H5N3)	99.35 (1684/1695) 99.29 (1683/1695) 99.29 (1683/1695)
NP	MT477770.1	EPI1567180 EPI1062334 EPI1062329	A/wild waterfowl/Korea/F14-5/2016 (A/H6N1) A/Bean goose/Hubei/CH-i177/2017_H7N7 (A/H7N7) A/Bean goose/Hubei/CH-I299/2017_H7N7 (A/H7N7)	99.60 (1491/1497) 99.60 (1491/1497) 99.60 (1491/1497)
NA	MT477771.1	EPI1513961 EPI1514049 EPI1514041	A/mallard/Korea/H15-1/2017 (A/H5N2) A/bean goose/Korea/H112/2017 (A/H5N2) A/spot-billed duck/Korea/H51/2017 (A/H5N2)	99.01 (1396/1410) 98.72 (1392/1410) 98.72 (1392/1410)
M	MT477772.1	EPI1567198 EPI1567190 EPI1098997	A/bean goose/Korea/F54-8/2017 (A/H6N1) A/bean goose/Korea/F27-6/2017 (A/H6N1) A/duck/Bangladesh/31227/2016 (A/H6N2)	99.80 (980/982) 99.80 (980/982) 99.80 (980/982)
NS	MT477773.1	EPI1848486 EPI1635085 EPI1635077	A/Environment/Guangxi/13425/2018 (A/H10N3) A/duck/Cambodia/C50W8M1/2018 (A/H7N4) A/duck/Cambodia/12T-24-1-D17/2018 (A/H7N4)	99.52 (834/838) 99.52 (834/838) 99.28 (832/838)

**Table 2:** Sequence homology of the whole A/Bean Goose/South Korea/KNU18-86/2018 (A/H5N2) genome.

Gene Segment	Genbank ID	Reference Strain Accession ID	Highest Similarly Reference Strain	Identity (%)
PB2	MT477790.1	EPI965204 EPI965285 EPI1332121	A/duck/Bangladesh/24697/2015 (A/H15N9) A/duck/Bangladesh/24704/2015 (A/H15N9) A/Environment/Jiangxi/22207/2014 (A/H4N2)	99.08 (2259/2280) 99.04 (2258/2280) 98.82 (2259/2280)
PB1	MT477791.1	EPI1549213 EPI1549229 EPI1549221	A/wild bird/Eastern China/1754/2017 (A/H5N3) A/wild bird/Eastern China/1759/2017 (A/H5N3) A/wild bird/Eastern China/1758/2017 (A/H5N3)	99.38 (2260/2274) 99.34 (2259/2274) 99.34 (2259/2274)
PA	MT477792.1	EPI1619612 EPI1513958 EPI1848485	A/Anser Fabalis/Jiangsu/J746/2017 (A/H6N1) A/mallard/Korea/H15-1/2017 (A/H5N2) A/Environment/Guangxi/13425/2018 (A/H10N3)	99.21 (2136/2153) 99.16 (2135/2153) 99.07 (2133/2153)
HA	MT477793.1	EPI1514074 EPI1513954 EPI1513946	A/spot-billed duck/Korea/H10-1/2017 (A/H5N3) A/mallard/Korea/A44-5/2017 (A/H5N3) A/mallard/Korea/A33-5/2017 (A/H5N2)	99.23 (1682/1695) 99.23 (1682/1695) 99.17 (1681/1695)
NP	MT477794.1	EPI1567180 EPI1062334 EPI1062329	A/wild waterfowl/Korea/F14-5/2016 (A/H6N1) A/Bean goose/Hubei/CH-i177/2017_H7N7 (A/H7N7) A/Bean goose/Hubei/CH-I299/2017_H7N7 (A/H7N7)	99.53 (1490/1497) 99.53 (1490/1497) 99.53 (1490/1497)
NA	MT477795.1	EPI1513961 EPI1514041 EPI1514049	A/mallard/Korea/H15-1/2017 (A/H5N2) A/spot-billed duck/Korea/H51/2017 (A/H5N2) A/bean goose/Korea/H112/2017 (A/H5N2)	99.22 (1399/1410) 98.65 (1391/1410) 98.58 (1390/1410)
M	MT477796.1	EPI1567198 EPI1567190 EPI1098997	A/bean goose/Korea/F54-8/2017 (A/H6N1) A/bean goose/Korea/F27-6/2017 (A/H6N1) A/duck/Bangladesh/31227/2016 (A/H6N2)	99.80 (980/982) 99.80 (980/982) 99.80 (980/982)
NS	MT477797.1	EPI1848486 EPI1635085 EPI1635077	A/Environment/Guangxi/13425/2018 (A/H10N3) A/duck/Cambodia/C50W8M1/2018 (A/H7N4) A/duck/Cambodia/12T-24-1-D17/2018 (A/H7N4)	99.64 (835/838) 99.64 (835/838) 99.40 (833/838)

**Table 3:** Sequence homology of the whole A/Wild duck/South Korea/KNU18-93/2018 (A/H5N2) genome.

Gene Segment	Genbank ID	Reference Strain Accession ID	Highest Similarly Reference Strain	Identity (%)
PB2	MT477798.1	EPI965204 EPI965285 EPI1332121	A/duck/Bangladesh/24697/2015 (A/H15N9) A/duck/Bangladesh/24704/2015 (A/H15N9) A/Environment/Jiangxi/22207/2014 (A/H4N2)	99.04 (2258/2280) 98.99 (2257/2280) 98.77 (2252/2280)
PB1	MT477799.1	EPI1549213 EPI1549229 EPI1549221	A/wild bird/Eastern China/1754/2017 (A/H5N3) A/wild bird/Eastern China/1759/2017 (A/H5N3) A/wild bird/Eastern China/1758/2017 (A/H5N3)	99.30 (2258/2274) 99.25 (2257/2274) 99.25 (2257/2274)
PA	MT477800.1	EPI1619612 EPI1513958 EPI867668	A/Anser Fabalis/Jiangsu/J746/2017 (A/H6N1) A/mallard/Korea/H15-1/2017 (A/H5N2) A/duck/Gunma/3/2016 (A/H3N8)	99.49 (2140/2151) 99.44 (2139/2151) 99.12 (2132/2151)
HA	MT477801.1	EPI1514074 EPI1513954 EPI1513946	A/spot-billed duck/Korea/H10-1/2017 (A/H5N3) A/mallard/Korea/A44-5/2017 (A/H5N3) A/mallard/Korea/A33-5/2017 (A/H5N2)	99.23 (1682/1695) 99.23 (1682/1695) 99.17 (1681/1695)
NP	MT477802.1	EPI1567180 EPI1062334 EPI1062329	A/wild waterfowl/Korea/F14-5/2016 (A/H6N1) A/Bean goose/Hubei/CH-i177/2017_H7N7 (A/H7N7) A/Bean goose/Hubei/CH-I299/2017_H7N7 (A/H7N7)	99.40 (1488/1497) 99.40 (1488/1497) 99.40 (1488/1497)
NA	MT477803.1	EPI1513961 EPI1514041 EPI1514049	A/mallard/Korea/H15-1/2017 (A/H5N2) A/spot-billed duck/Korea/H51/2017 (A/H5N2) A/bean goose/Korea/H112/2017 (A/H5N2)	98.94 (1395/1410) 98.37 (1387/1410) 98.30 (1386/1410)
M	MT477804.1	EPI1567198 EPI1567190 EPI1098997	A/bean goose/Korea/F54-8/2017 (A/H6N1) A/bean goose/Korea/F27-6/2017 (A/H6N1) A/duck/Bangladesh/31227/2016 (A/H6N2)	99.80 (980/982) 99.80 (980/982) 99.80 (980/982)
NS	MT477805.1	EPI1521597 EPI1619617 EPI1567328	A/duck/Hokkaido/56/2017 (A/H12N2) A/Anser Fabalis/Jiangsu/J746/2017 (A/H6N1) A/whooper swan/Iwate/21/2017 (A/H6N8)	99.76 (836/838) 99.64 (836/838) 99.64 (836/838)

**Table 4:** Comparison between the hemagglutinin (HA) receptor-binding sites and neuraminidase (NA) of the three novel avian influenza H5N2 viruses and those of low pathogenic and highly pathogenic H5 viruses.

Virus Strain	HA Receptor-Binding Residues (H5 Numbering)									NA				
	Cleavage Sites	D/E94N	I116M	S121N	A134V	G139R	S142G	D221G/N	Q222L	Deleted Range from 50–70	M26I	I106V	T223I	K/S373A/N
KNU18-28 (H5N2)	PQRETR↓GLF	D	I	S	A	G	S	G	Q	No deletion	I	I	I	S
KNU18-86 (H5N2)	PQRETR↓GLF	D	I	S	A	G	S	G	Q	No deletion	I	I	I	S
KNU18-93 (H5N2)	PQRETR↓GLF	D	I	S	A	G	S	G	Q	No deletion	I	I	I	S
KNU18-91 (H5N3)	PQRETR↓GLF	D	I	S	A	G	S	G	Q	No deletion	I	I	I	S
KA14 (H5N2)	PQRETR↓GLF	D	I	S	A	G	S	G	Q	No deletion	I	I	I	S
Z7450 (H5N2)	RERRRKR↓GLF	T	I	S	A	G	S	G	Q	No deletion	I	I	I	S

KNU18-28: A/Wild duck/South Korea/KNU18-28/2018 (H5N2), KNU18-86: A/Bean Goose/South Korea/KNU18-86/2018 (H5N2), KNU18-93: A/Wild duck/South Korea/KNU18-93/2018, KNU18-91: A/mallard duck/South Korea/KNU18-91/2018 (H5N3) LPAI, KA14: A/duck/Korea/A14/2008 (H5N2) LPAI, and Z7450: A/chicken/Zhejiang/7450/2015 (H5N2) HPAI.

**Table 5:** Amino acid mutation analysis of eight gene segments responsible for enhancing polymerase activity, viral transmissibility, and virulence of the three novel H5N2 viruses.

Viral Protein	Amino Acid	KA14	Z7450	KNU18-91	KNU18-28	KNU18-86	KNU18-93	Phenotype	References
PB2	L89V	V	V	V	V	V	V	Enhanced polymerase activity, increased virulence in mice	[26]
	K251R	R	R	R	R	R	R	Increased virulence in mice	[27]
	T309D	D	D	D	D	D	D	Enhanced polymerase activity, increased virulence in mice	[26]
	T339K	K	K	K	K	K	K	Enhanced polymerase activity, increased virulence in mice	[26]
	Q368R	R	R	R	R	R	R	Increased polymerase activity, increased virulence in mammals	[28]
	H447Q	Q	Q	Q	Q	Q	Q	Increased polymerase activity, increased virulence in mammals	[28]
	R477G	G	G	G	G	G	G	Enhanced polymerase activity, increased virulence in mice	[26]
	I495V	V	V	V	V	V	V	Enhanced polymerase activity, increased virulence in mice	[26]
	A676T	T	T	T	T	T	T	Enhanced polymerase active, increased virulence in mice	[26]
PB1	D/A3V	V	V	V	V	V	V	Increased polymerase activity, increased virulence in mammals	[28,29]
	L13P	P	P	P	P	P	P	Increased polymerase activity, increased virulence in mammals	[30]
	R207K	K	K	K	K	K	K	Increased polymerase activity in mammalian cells	[31]
	K328N	N	N	N	N	N	N	Increased polymerase activity, increased virulence in mammals	[28,29]
	H436Y	Y	Y	Y	Y	Y	Y	Increased polymerase activity and virulence in mallards, ferrets, and mice	[31]
	A469T	T	T	T	T	T	T	Conferred in contact transmissibility in guinea pigs	[32]
	L473V	V	V	V	V	V	V	Increased polymerase activity and replication efficiency	[33]
	V652A	A	A	A	A	A	A	Increased virulence in mice	[27]
PA	S37A	A	A	A	A	A	A	Significantly increased viral growth and polymerase activity in mammalian cells	[34]
	H266R	R	R	R	R	R	R	Increased polymerase activity, increased virulence in mammals and birds	[35,36]
	F277S	S	S	S	S	S	S	Contributed to the virulence and mammalian adaptation	[36]
	C278Q	Q	Q	Q	Q	Q	Q	Adapt to mammalian hosts	[37]
	S/A515T	T	T	T	T	T	T	Increased polymerase activity, increased virulence in mammals and birds	[31]
HA	A/I/P/S/T86V	A	T	V	V	V	V	Increased virulence in mammals	[38,39]
	Q/H/I138L/N	Q	N	N	N	N	N	Increased virulence in mammals	[38,39]
	K212E/R/G	K	E	E	E	E	E	Increased virulence in mammals	[38,39]
	G395E	E	E	E	E	E	E	Enhanced polymerase activity, increased virulence in mice	[40]
	F427L	L	L	L	L	L	L	Important for adaptation of H5N5 AIVs to mammals	[41]
NP	V41I	I	I	I	I	I	I	Might contribute to viral transmissibility	[42]
	V105M	M	M	M	M	M	M	Contribute to the increased virulence of the H9N2	[43]
	D210E	E	E	E	E	E	E	Might contribute to viral transmissibility	[42]
	F253I	I	I	I	I	I	I	Results in attenuated pathogenicity of the virus in mice	[44]
	I353V	V	V	V	V	V	I	Increased virulence in mice	[27]
NA	M26I	I	I	I	I	I	I	Increased virulence in mice	[24]
	R143K	R	K	K	K	K	K	Increased virulence in mammals and mice	[45]
	T223I	I	I	I	I	I	I	Increased virulence in mammals	[25,46,47]
M1	N30D	D	D	D	D	D	D	Increased virulence in mammals	[48]
	A166V	A	V	V	V	V	V	Contribute to the increased virulence of the H9N2.	[43]
NS1	A/P42S	S	S	A	S	S	S	Increased virulence in mammals, antagonism of IFN induction	[38]
	T/D/V/R/A127N	N	N	R	N	N	N	Increased virulence in mammals	[38]
	V149A	A	A	A	A	A	A	Pathogenicity in mice, antagonism of IFN induction	[49]

KNU18-28: A/Wild duck/South Korea/KNU18-28/2018 (H5N2), KNU18-86: A/Bean Goose/South Korea/KNU18-86/2018 (H5N2), KNU18-93: A/Wild duck/South Korea/KNU18-93/2018, KNU18-91: A/mallard duck/South Korea/KNU18-91/2018 (H5N3) LPAI, KA14: A/duck/Korea/A14/2008 (H5N2) LPAI, and Z7450: A/chicken/Zhejiang/7450/2015 (H5N2) HPAI.

## Data Availability

All the data presented in this study are available in this article and Supplementary Materials.

## Supplementary Materials

The following are available online at https://www.mdpi.com/article/10.3390/v13112192/s1, Figure S1. Phylogenetic tree analysis of gene segments based on the nucleotide sequences. Figure S2. Raw ELISA data of TCID_50_ assay to determine the viral growth kinetics in MDCK cells. Figure S3. Raw ELISA data of TCID_50_ assay for viral load shedding in infected mouse lung. Figure S4. Lungs from mouse infected H1N1, H5N3, KNU18-28, KNU18-86, and KNU18-93 at 6 day post-infection. Table S1. Genome identity comparison of three novel H5N2 (KNU18-28, KNU18-86, and KNU18-93) isolates and H5N3 (KNU18-91) isolate.
