# Supplementary material for: Genetic Characterization and Pathogenesis of Three Novel Reassortant H5N2 Viruses in South Korea, 2018
Source: Viruses. 2021 Oct 30;13(11):2192. doi: 10.3390/v13112192 (PMC8619638; doi:10.3390/v13112192)
Supplement: Supplementary file 1 [file viruses-13-02192-s001.zip › viruses-1432543-supplementary.pdf]

Supplementary Materials

Genetic Characterization and Pathogenesis of Three Novel Reassortant H5N2 Viruses in South Korea, 2018

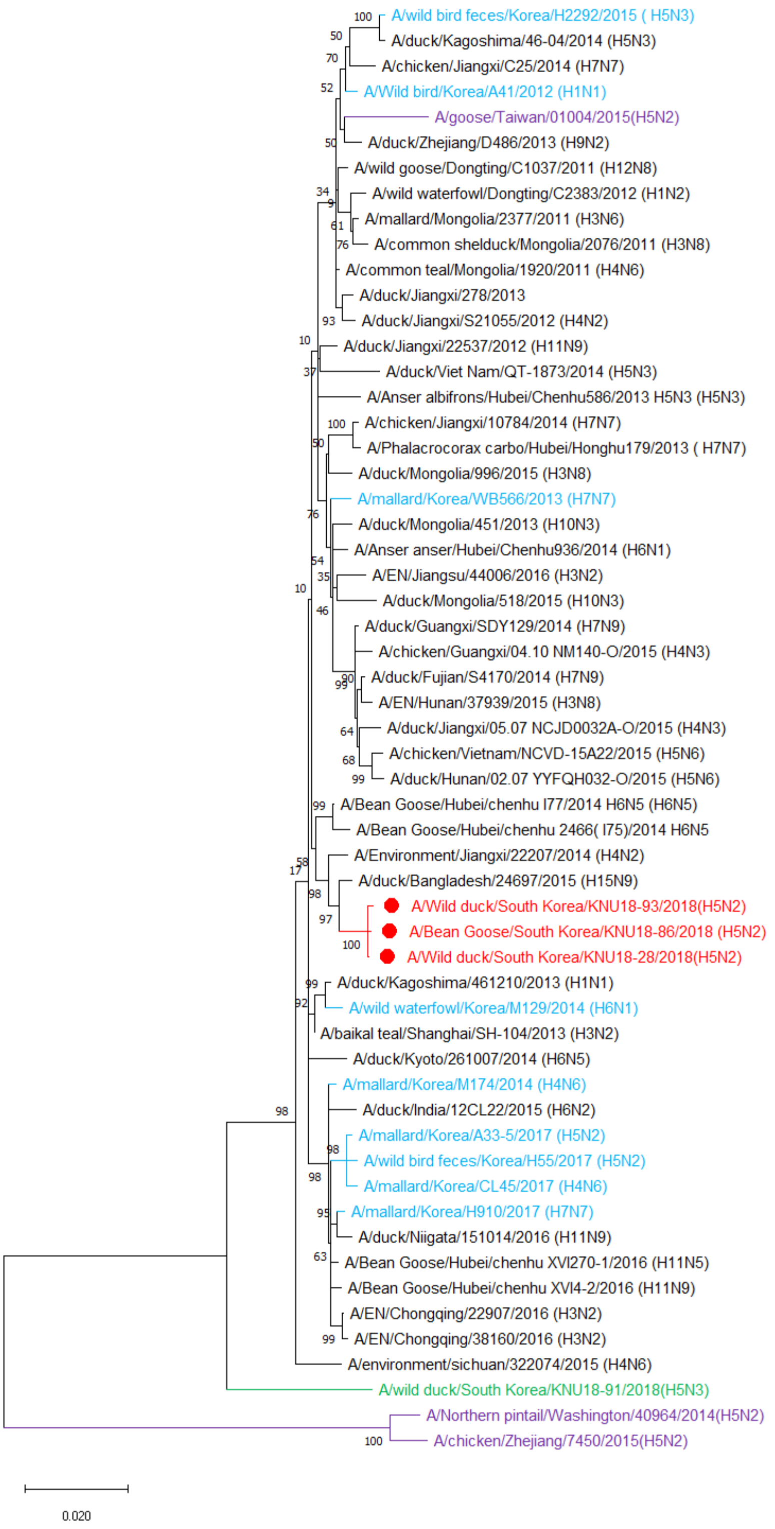

(**a**) PB2

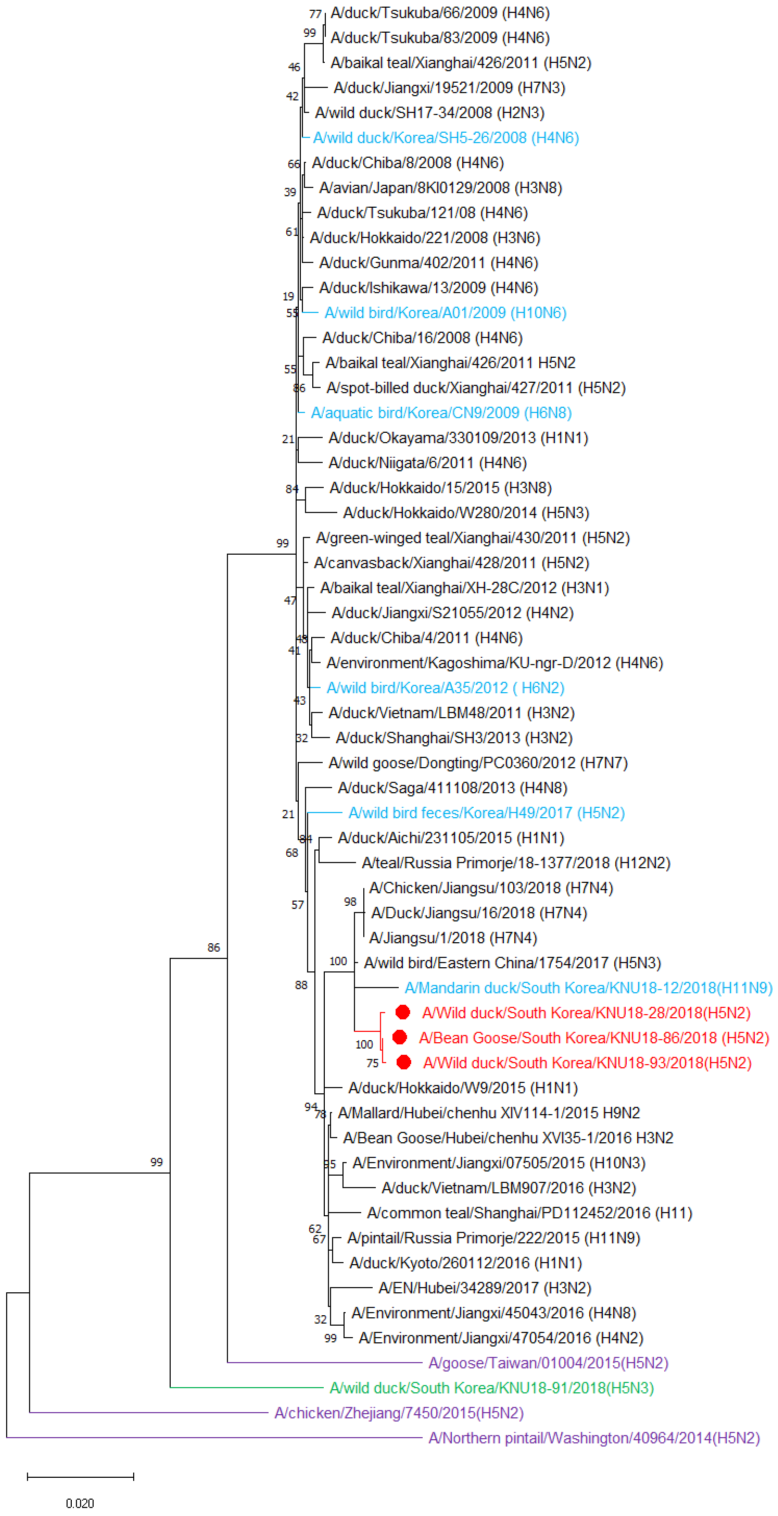
A/duck/Tsukuba/66/2009 (H4N6)
A/duck/Tsukuba/83/2009 (H4N6)
A/baikal teal/Xianghai/426/2011 (H5N2)
A/duck/Jiangxi/19521/2009 (H7N3)
A/wild duck/SH17-34/2008 (H2N3)
A/wild duck/Korea/SH5-26/2008 (H4N6)
A/duck/Chiba/8/2008 (H4N6)
A/avian/Japan/8KI0129/2008 (H3N8)
A/duck/Tsukuba/121/08 (H4N6)
A/duck/Hokkaido/221/2008 (H3N6)
A/duck/Gunma/402/2011 (H4N6)
A/duck/Ishikawa/13/2009 (H4N6)
A/wild bird/Korea/A01/2009 (H10N6)
A/duck/Chiba/16/2008 (H4N6)
A/baikal teal/Xianghai/426/2011 H5N2
A/spot-billed duck/Xianghai/427/2011 (H5N2)
A/aquatic bird/Korea/CN9/2009 (H6N8)
A/duck/Okayama/330109/2013 (H1N1)
A/duck/Niigata/6/2011 (H4N6)
A/duck/Hokkaido/15/2015 (H3N8)
A/duck/Hokkaido/W280/2014 (H5N3)
A/green-winged teal/Xianghai/430/2011 (H5N2)
A/canvasback/Xianghai/428/2011 (H5N2)
A/baikal teal/Xianghai/XH-28C/2012 (H3N1)
A/duck/Jiangxi/S21055/2012 (H4N2)
A/duck/Chiba/4/2011 (H4N6)
A/environment/Kagoshima/KU-ngr-D/2012 (H4N6)
A/wild bird/Korea/A35/2012 (H6N2)
A/duck/Vietnam/LBM48/2011 (H3N2)
A/duck/Shanghai/SH3/2013 (H3N2)
A/wild goose/Dongting/PC0360/2012 (H7N7)
A/duck/Saga/411108/2013 (H4N8)
A/wild bird feces/Korea/H49/2017 (H5N2)
A/duck/Aichi/231105/2015 (H1N1)
A/teal/Russia Primorje/18-1377/2018 (H12N2)
A/Chicken/Jiangsu/103/2018 (H7N4)
A/Duck/Jiangsu/16/2018 (H7N4)
A/Jiangsu/1/2018 (H7N4)
A/wild bird/Eastern China/1754/2017 (H5N3)
A/Mandarin duck/South Korea/KNU18-12/2018(H11N9)
A/Wild duck/South Korea/KNU18-28/2018(H5N2)
A/Bean Goose/South Korea/KNU18-86/2018 (H5N2)
A/Wild duck/South Korea/KNU18-93/2018(H5N2)
A/duck/Hokkaido/W9/2015 (H1N1)
A/Mallard/Hubei/chenhu XIV114-1/2015 H9N2
A/Bean Goose/Hubei/chenhu XVI35-1/2016 H3N2
A/Environment/Jiangxi/07505/2015 (H10N3)
A/duck/Vietnam/LBM907/2016 (H3N2)
A/common teal/Shanghai/PD112452/2016 (H11)
A/pintail/Russia Primorje/222/2015 (H11N9)
A/duck/Kyoto/260112/2016 (H1N1)
A/EN/Hubei/34289/2017 (H3N2)
A/Environment/Jiangxi/45043/2016 (H4N8)
A/Environment/Jiangxi/47054/2016 (H4N2)
A/goose/Taiwan/01004/2015(H5N2)
A/wild duck/South Korea/KNU18-91/2018(H5N3)
A/chicken/Zhejiang/7450/2015(H5N2)
A/Northern pintail/Washington/40964/2014(H5N2)
0.020

(**b**) PB1

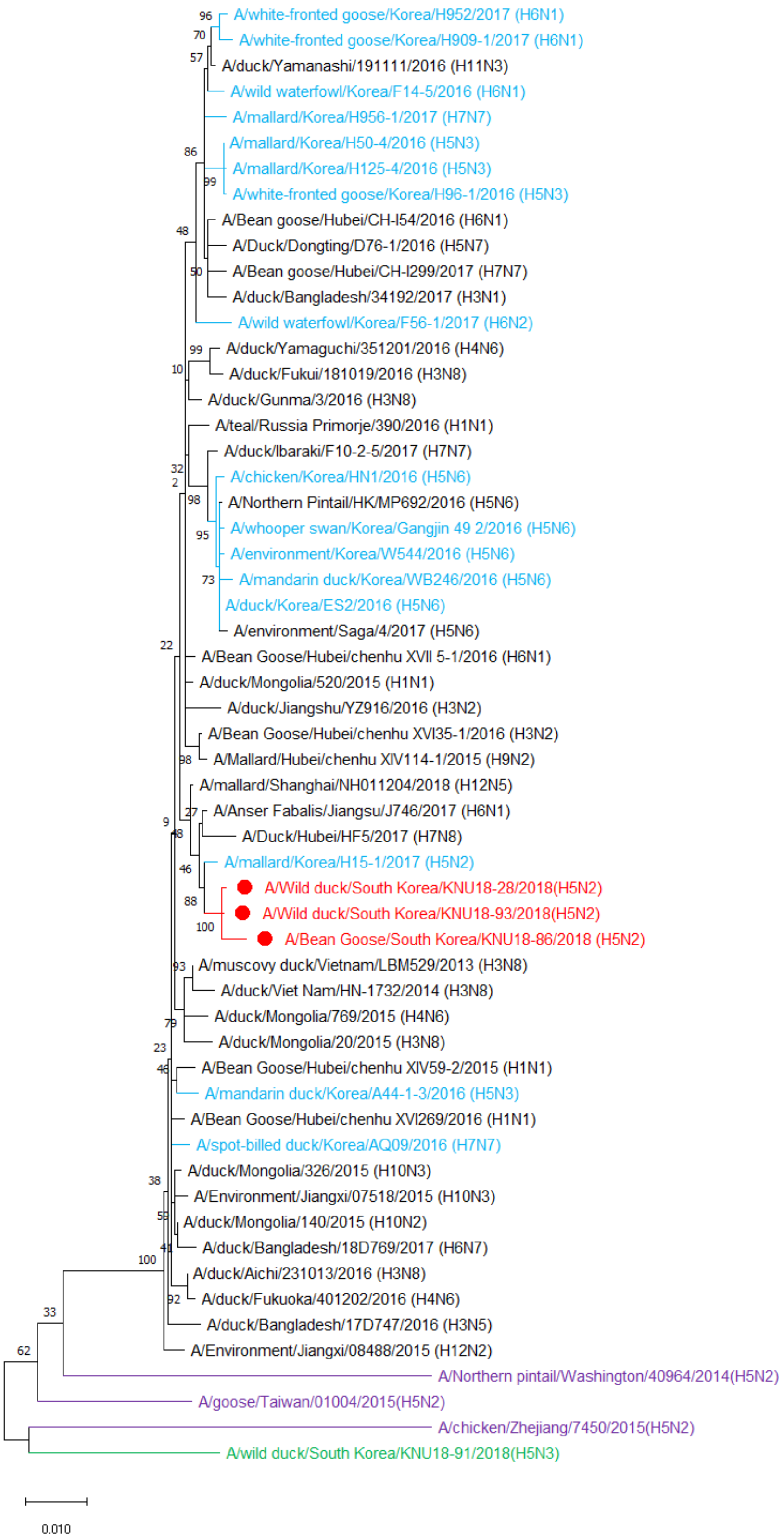
A/white-fronted goose/Korea/H952/2017 (H6N1)
A/white-fronted goose/Korea/H909-1/2017 (H6N1)
A/duck/Yamanashi/191111/2016 (H11N3)
A/wild waterfowl/Korea/F14-5/2016 (H6N1)
A/mallard/Korea/H956-1/2017 (H7N7)
A/mallard/Korea/H50-4/2016 (H5N3)
A/mallard/Korea/H125-4/2016 (H5N3)
A/white-fronted goose/Korea/H96-1/2016 (H5N3)
A/Bean goose/Hubei/CH-I54/2016 (H6N1)
A/Duck/Dongting/D76-1/2016 (H5N7)
A/Bean goose/Hubei/CH-I299/2017 (H7N7)
A/duck/Bangladesh/34192/2017 (H3N1)
A/wild waterfowl/Korea/F56-1/2017 (H6N2)
A/duck/Yamaguchi/351201/2016 (H4N6)
A/duck/Fukui/181019/2016 (H3N8)
A/duck/Gunma/3/2016 (H3N8)
A/teal/Russia Primorje/390/2016 (H1N1)
A/duck/Ibaraki/F10-2-5/2017 (H7N7)
A/chicken/Korea/HN1/2016 (H5N6)
A/Northern Pintail/HK/MP692/2016 (H5N6)
A/whooper swan/Korea/Gangjin 49 2/2016 (H5N6)
A/environment/Korea/W544/2016 (H5N6)
A/mandarin duck/Korea/WB246/2016 (H5N6)
A/duck/Korea/ES2/2016 (H5N6)
A/environment/Saga/4/2017 (H5N6)
A/Bean Goose/Hubei/chenhu XVII 5-1/2016 (H6N1)
A/duck/Mongolia/520/2015 (H1N1)
A/duck/Jiangshu/YZ916/2016 (H3N2)
A/Bean Goose/Hubei/chenhu XVI35-1/2016 (H3N2)
A/Mallard/Hubei/chenhu XIV114-1/2015 (H9N2)
A/mallard/Shanghai/NH011204/2018 (H12N5)
A/Anser Fabalis/Jiangsu/J746/2017 (H6N1)
A/Duck/Hubei/HF5/2017 (H7N8)
A/mallard/Korea/H15-1/2017 (H5N2)
A/Wild duck/South Korea/KNU18-28/2018(H5N2)
A/Wild duck/South Korea/KNU18-93/2018(H5N2)
A/Bean Goose/South Korea/KNU18-86/2018 (H5N2)
A/muscovy duck/Vietnam/LBM529/2013 (H3N8)
A/duck/Viet Nam/HN-1732/2014 (H3N8)
A/duck/Mongolia/769/2015 (H4N6)
A/duck/Mongolia/20/2015 (H3N8)
A/Bean Goose/Hubei/chenhu XIV59-2/2015 (H1N1)
A/mandarin duck/Korea/A44-1-3/2016 (H5N3)
A/Bean Goose/Hubei/chenhu XVI269/2016 (H1N1)
A/spot-billed duck/Korea/AQ09/2016 (H7N7)
A/duck/Mongolia/326/2015 (H10N3)
A/Environment/Jiangxi/07518/2015 (H10N3)
A/duck/Mongolia/140/2015 (H10N2)
A/duck/Bangladesh/18D769/2017 (H6N7)
A/duck/Aichi/231013/2016 (H3N8)
A/duck/Fukuoka/401202/2016 (H4N6)
A/duck/Bangladesh/17D747/2016 (H3N5)
A/Environment/Jiangxi/08488/2015 (H12N2)
A/Northern pintail/Washington/40964/2014(H5N2)
A/goose/Taiwan/01004/2015(H5N2)
A/chicken/Zhejiang/7450/2015(H5N2)
A/wild duck/South Korea/KNU18-91/2018(H5N3)
0.010

(**c**) PA

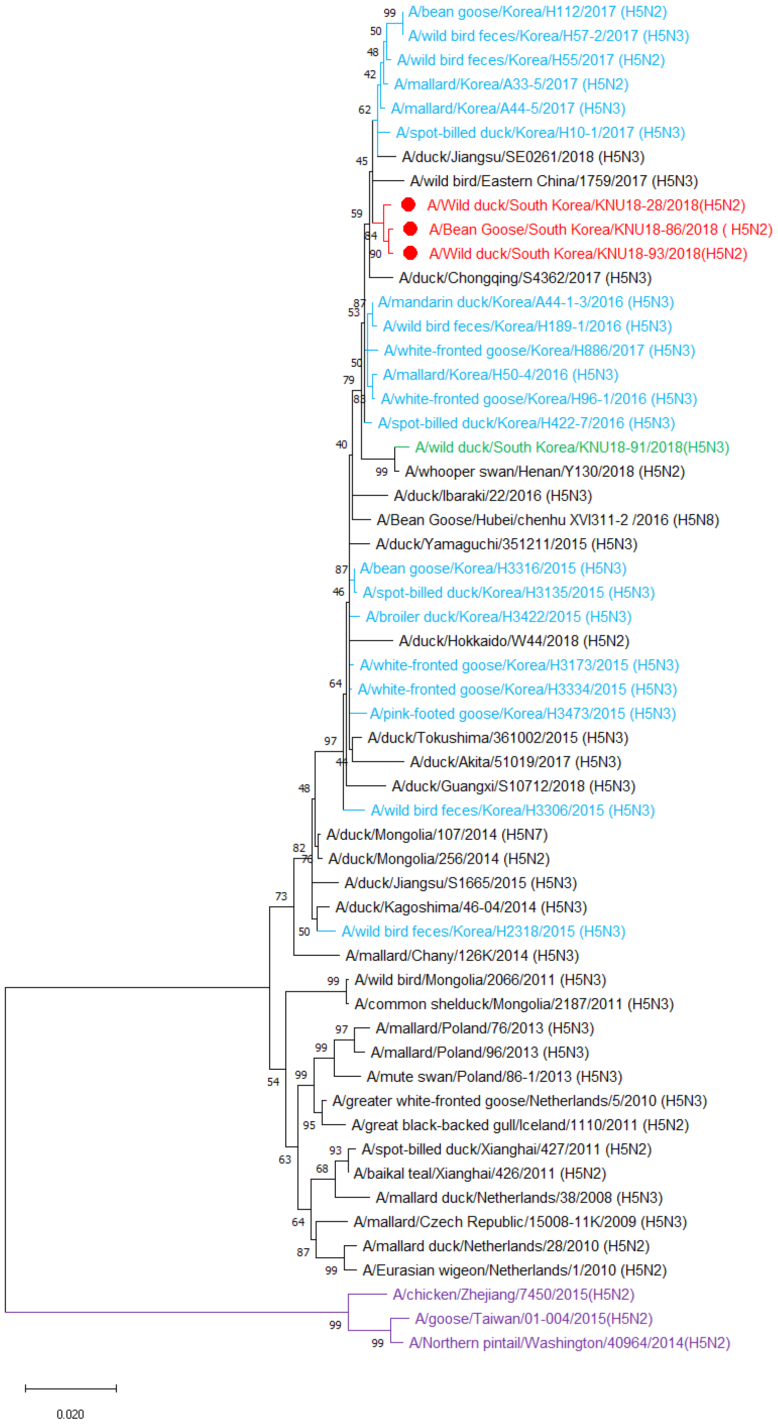
A/bean goose/Korea/H112/2017 (H5N2)
A/wild bird feces/Korea/H57-2/2017 (H5N3)
A/wild bird feces/Korea/H55/2017 (H5N2)
A/mallard/Korea/A33-5/2017 (H5N2)
A/mallard/Korea/A44-5/2017 (H5N3)
A/spot-billed duck/Korea/H10-1/2017 (H5N3)
A/duck/Jiangsu/SE0261/2018 (H5N3)
A/wild bird/Eastern China/1759/2017 (H5N3)
A/Wild duck/South Korea/KNU18-28/2018(H5N2)
A/Bean Goose/South Korea/KNU18-86/2018 (H5N2)
A/Wild duck/South Korea/KNU18-93/2018(H5N2)
A/duck/Chongqing/S4362/2017 (H5N3)
A/mandarin duck/Korea/A44-1-3/2016 (H5N3)
A/wild bird feces/Korea/H189-1/2016 (H5N3)
A/white-fronted goose/Korea/H886/2017 (H5N3)
A/mallard/Korea/H50-4/2016 (H5N3)
A/white-fronted goose/Korea/H96-1/2016 (H5N3)
A/spot-billed duck/Korea/H422-7/2016 (H5N3)
A/wild duck/South Korea/KNU18-91/2018(H5N3)
A/whooper swan/Henan/Y130/2018 (H5N2)
A/duck/Ibaraki/22/2016 (H5N3)
A/Bean Goose/Hubei/chenhu XVI311-2 /2016 (H5N8)
A/duck/Yamaguchi/351211/2015 (H5N3)
A/bean goose/Korea/H3316/2015 (H5N3)
A/spot-billed duck/Korea/H3135/2015 (H5N3)
A/broiler duck/Korea/H3422/2015 (H5N3)
A/duck/Hokkaido/W44/2018 (H5N2)
A/white-fronted goose/Korea/H3173/2015 (H5N3)
A/white-fronted goose/Korea/H3334/2015 (H5N3)
A/pink-footed goose/Korea/H3473/2015 (H5N3)
A/duck/Tokushima/361002/2015 (H5N3)
A/duck/Akita/51019/2017 (H5N3)
A/duck/Guangxi/S10712/2018 (H5N3)
A/wild bird feces/Korea/H3306/2015 (H5N3)
A/duck/Mongolia/107/2014 (H5N7)
A/duck/Mongolia/256/2014 (H5N2)
A/duck/Jiangsu/S1665/2015 (H5N3)
A/duck/Kagoshima/46-04/2014 (H5N3)
A/wild bird feces/Korea/H2318/2015 (H5N3)
A/mallard/Chany/126K/2014 (H5N3)
A/wild bird/Mongolia/2066/2011 (H5N3)
A/common shelduck/Mongolia/2187/2011 (H5N3)
A/mallard/Poland/76/2013 (H5N3)
A/mallard/Poland/96/2013 (H5N3)
A/mute swan/Poland/86-1/2013 (H5N3)
A/greater white-fronted goose/Netherlands/5/2010 (H5N3)
A/great black-backed gull/Iceland/1110/2011 (H5N2)
A/spot-billed duck/Xianghai/427/2011 (H5N2)
A/baikal teal/Xianghai/426/2011 (H5N2)
A/mallard duck/Netherlands/38/2008 (H5N3)
A/mallard/Czech Republic/15008-11K/2009 (H5N3)
A/mallard duck/Netherlands/28/2010 (H5N2)
A/Eurasian wigeon/Netherlands/1/2010 (H5N2)
A/chicken/Zhejiang/7450/2015(H5N2)
A/goose/Taiwan/01-004/2015(H5N2)
A/Northern pintail/Washington/40964/2014(H5N2)
0.020

(**d**) HA

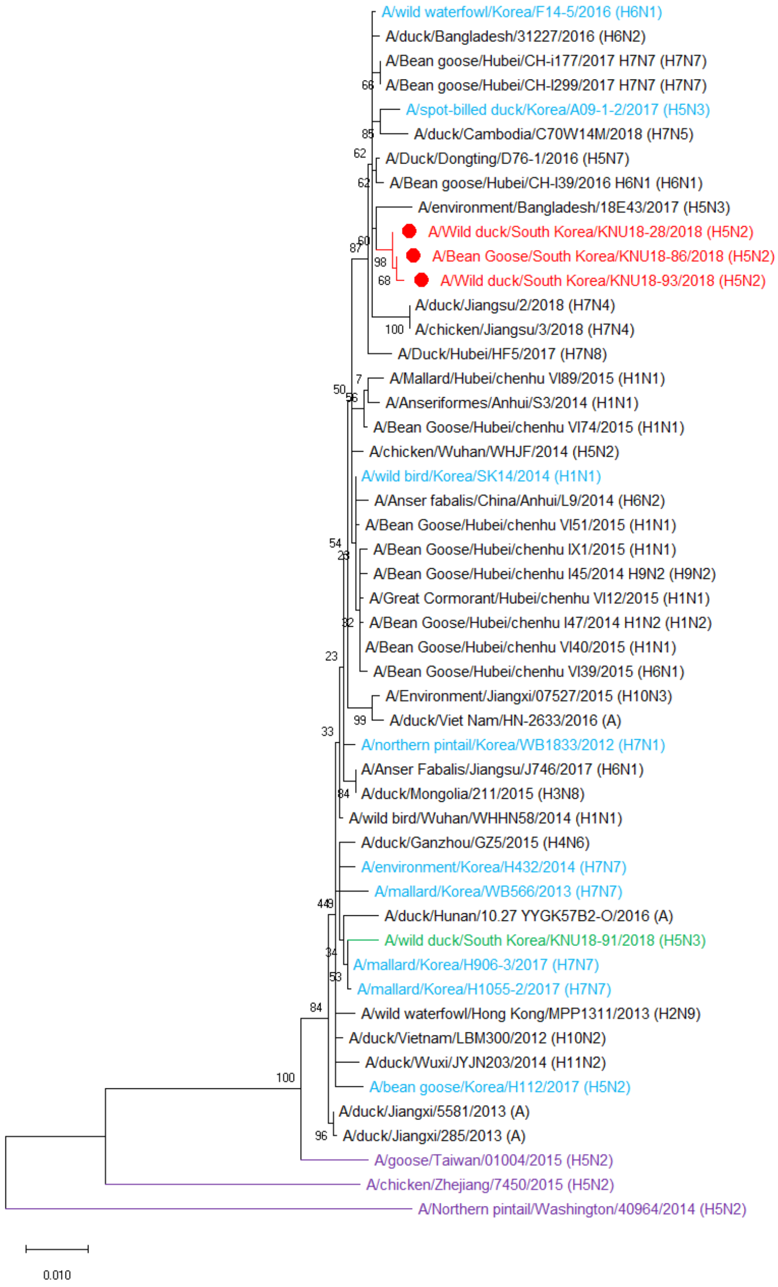
A/wild waterfowl/Korea/F14-5/2016 (H6N1)
A/duck/Bangladesh/31227/2016 (H6N2)
A/Bean goose/Hubei/CH-i177/2017 H7N7 (H7N7)
A/Bean goose/Hubei/CH-I299/2017 H7N7 (H7N7)
A/spot-billed duck/Korea/A09-1-2/2017 (H5N3)
A/duck/Cambodia/C70W14M/2018 (H7N5)
A/Duck/Dongting/D76-1/2016 (H5N7)
A/Bean goose/Hubei/CH-I39/2016 H6N1 (H6N1)
A/environment/Bangladesh/18E43/2017 (H5N3)
A/Wild duck/South Korea/KNU18-28/2018 (H5N2)
A/Bean Goose/South Korea/KNU18-86/2018 (H5N2)
A/Wild duck/South Korea/KNU18-93/2018 (H5N2)
A/duck/Jiangsu/2/2018 (H7N4)
A/chicken/Jiangsu/3/2018 (H7N4)
A/Duck/Hubei/HF5/2017 (H7N8)
A/Mallard/Hubei/chenhu VI89/2015 (H1N1)
A/Anseriformes/Anhui/S3/2014 (H1N1)
A/Bean Goose/Hubei/chenhu VI74/2015 (H1N1)
A/chicken/Wuhan/WHJF/2014 (H5N2)
A/wild bird/Korea/SK14/2014 (H1N1)
A/Anser fabalis/China/Anhui/L9/2014 (H6N2)
A/Bean Goose/Hubei/chenhu VI51/2015 (H1N1)
A/Bean Goose/Hubei/chenhu IX1/2015 (H1N1)
A/Bean Goose/Hubei/chenhu I45/2014 H9N2 (H9N2)
A/Great Cormorant/Hubei/chenhu VI12/2015 (H1N1)
A/Bean Goose/Hubei/chenhu I47/2014 H1N2 (H1N2)
A/Bean Goose/Hubei/chenhu VI40/2015 (H1N1)
A/Bean Goose/Hubei/chenhu VI39/2015 (H6N1)
A/Environment/Jiangxi/07527/2015 (H10N3)
A/duck/Viet Nam/HN-2633/2016 (A)
A/northern pintail/Korea/WB1833/2012 (H7N1)
A/Anser Fabalis/Jiangsu/J746/2017 (H6N1)
A/duck/Mongolia/211/2015 (H3N8)
A/wild bird/Wuhan/WHHN58/2014 (H1N1)
A/duck/Ganzhou/GZ5/2015 (H4N6)
A/environment/Korea/H432/2014 (H7N7)
A/mallard/Korea/WB566/2013 (H7N7)
A/duck/Hunan/10.27 YYGK57B2-O/2016 (A)
A/wild duck/South Korea/KNU18-91/2018 (H5N3)
A/mallard/Korea/H906-3/2017 (H7N7)
A/mallard/Korea/H1055-2/2017 (H7N7)
A/wild waterfowl/Hong Kong/MPP1311/2013 (H2N9)
A/duck/Vietnam/LBM300/2012 (H10N2)
A/duck/Wuxi/JYJN203/2014 (H11N2)
A/bean goose/Korea/H112/2017 (H5N2)
A/duck/Jiangxi/5581/2013 (A)
A/duck/Jiangxi/285/2013 (A)
A/goose/Taiwan/01004/2015 (H5N2)
A/chicken/Zhejiang/7450/2015 (H5N2)
A/Northern pintail/Washington/40964/2014 (H5N2)
0.010

(e) NP

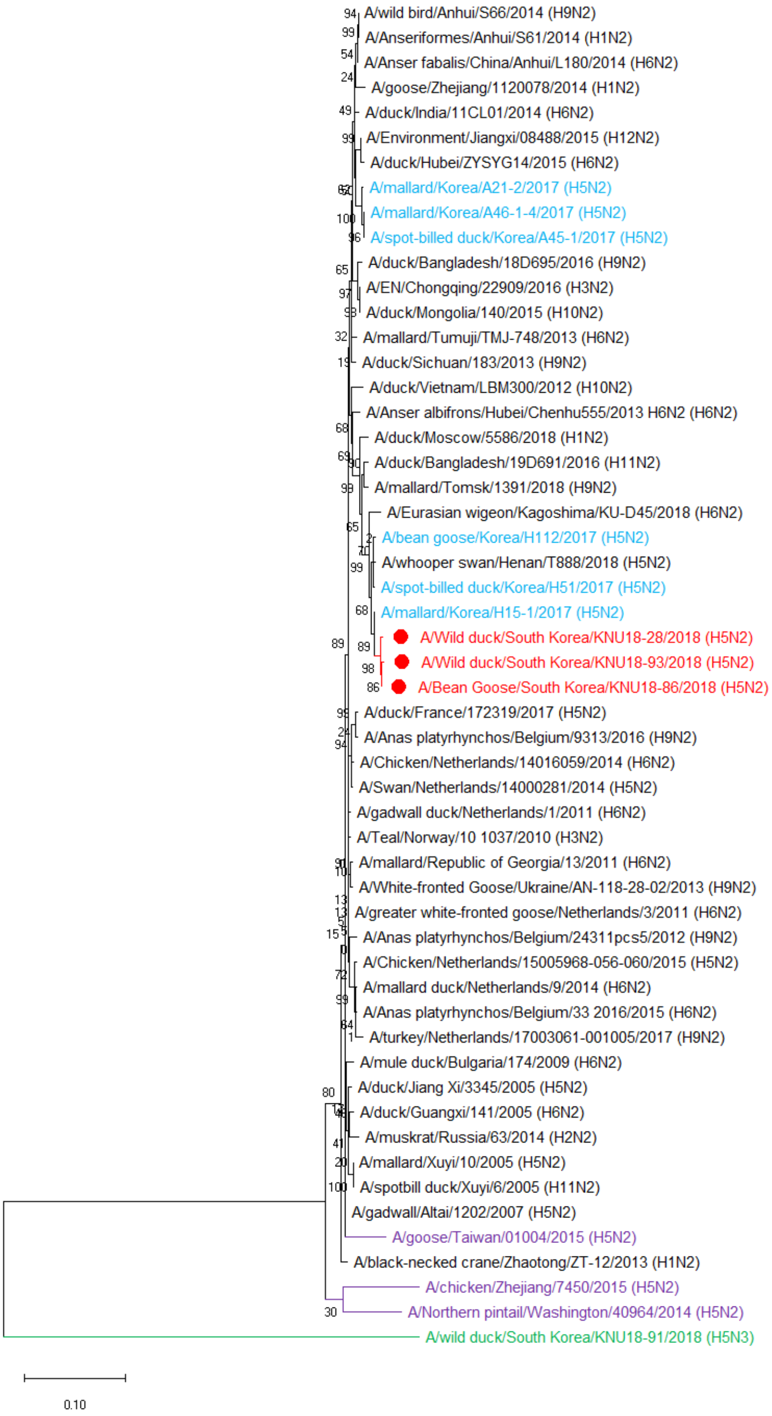
A/wild bird/Anhui/S66/2014 (H9N2)
A/Anseriformes/Anhui/S61/2014 (H1N2)
A/Anser fabalis/China/Anhui/L180/2014 (H6N2)
A/goose/Zhejiang/1120078/2014 (H1N2)
A/duck/India/11CL01/2014 (H6N2)
A/Environment/Jiangxi/08488/2015 (H12N2)
A/duck/Hubei/ZYSYG14/2015 (H6N2)
A/mallard/Korea/A21-2/2017 (H5N2)
A/mallard/Korea/A46-1-4/2017 (H5N2)
A/spot-billed duck/Korea/A45-1/2017 (H5N2)
A/duck/Bangladesh/18D695/2016 (H9N2)
A/EN/Chongqing/22909/2016 (H3N2)
A/duck/Mongolia/140/2015 (H10N2)
A/mallard/Tumuji/TMJ-748/2013 (H6N2)
A/duck/Sichuan/183/2013 (H9N2)
A/duck/Vietnam/LBM300/2012 (H10N2)
A/Anser albifrons/Hubei/Chenhu555/2013 H6N2 (H6N2)
A/duck/Moscow/5586/2018 (H1N2)
A/duck/Bangladesh/19D691/2016 (H11N2)
A/mallard/Tomsk/1391/2018 (H9N2)
A/Eurasian wigeon/Kagoshima/KU-D45/2018 (H6N2)
A/bean goose/Korea/H112/2017 (H5N2)
A/whooper swan/Henan/T888/2018 (H5N2)
A/spot-billed duck/Korea/H51/2017 (H5N2)
A/mallard/Korea/H15-1/2017 (H5N2)
A/Wild duck/South Korea/KNU18-28/2018 (H5N2)
A/Wild duck/South Korea/KNU18-93/2018 (H5N2)
A/Bean Goose/South Korea/KNU18-86/2018 (H5N2)
A/duck/France/172319/2017 (H5N2)
A/Anas platyrhynchos/Belgium/9313/2016 (H9N2)
A/Chicken/Netherlands/14016059/2014 (H6N2)
A/Swan/Netherlands/14000281/2014 (H5N2)
A/gadwall duck/Netherlands/1/2011 (H6N2)
A/Teal/Norway/10 1037/2010 (H3N2)
A/mallard/Republic of Georgia/13/2011 (H6N2)
A/White-fronted Goose/Ukraine/AN-118-28-02/2013 (H9N2)
A/greater white-fronted goose/Netherlands/3/2011 (H6N2)
A/Anas platyrhynchos/Belgium/24311pcs5/2012 (H9N2)
A/Chicken/Netherlands/15005968-056-060/2015 (H5N2)
A/mallard duck/Netherlands/9/2014 (H6N2)
A/Anas platyrhynchos/Belgium/33 2016/2015 (H6N2)
A/turkey/Netherlands/17003061-001005/2017 (H9N2)
A/mule duck/Bulgaria/174/2009 (H6N2)
A/duck/Jiang Xi/3345/2005 (H5N2)
A/duck/Guangxi/141/2005 (H6N2)
A/muskrat/Russia/63/2014 (H2N2)
A/mallard/Xuyi/10/2005 (H5N2)
A/spotbill duck/Xuyi/6/2005 (H11N2)
A/gadwall/Altai/1202/2007 (H5N2)
A/goose/Taiwan/01004/2015 (H5N2)
A/black-necked crane/Zhaotong/ZT-12/2013 (H1N2)
A/chicken/Zhejiang/7450/2015 (H5N2)
A/Northern pintail/Washington/40964/2014 (H5N2)
A/wild duck/South Korea/KNU18-91/2018 (H5N3)
0.10

(**f**) NA

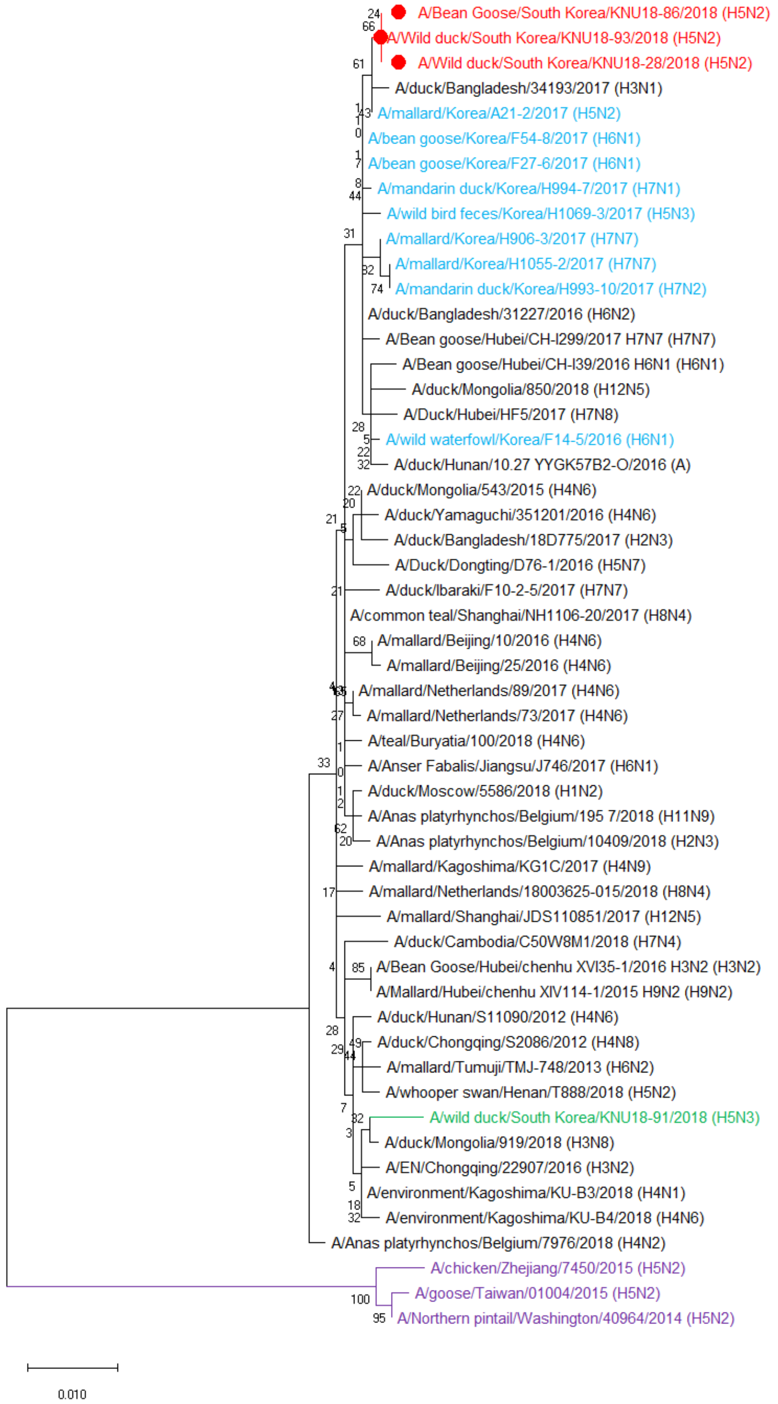

(**g**) M

Figure S1. Phylogenetic tree analysis of gene segments based on the nucleotide sequences. MEGA-X software using maximum likelihood method with bootstrap replication (1000 bootstraps) was used to generate the phylogenetic tree of PB2 (**a**), PB1 (**b**), PA (**c**), HA (**d**), NP (**e**), NA (**f**), and M (**g**). Red color: novel H5N2 isolates, blue color: Korean strains, purple color: H5N2 highly pathogenic avian influenza viruses (HPAIVs), green color: H5N3 strain isolated at the same surveillance time.

A

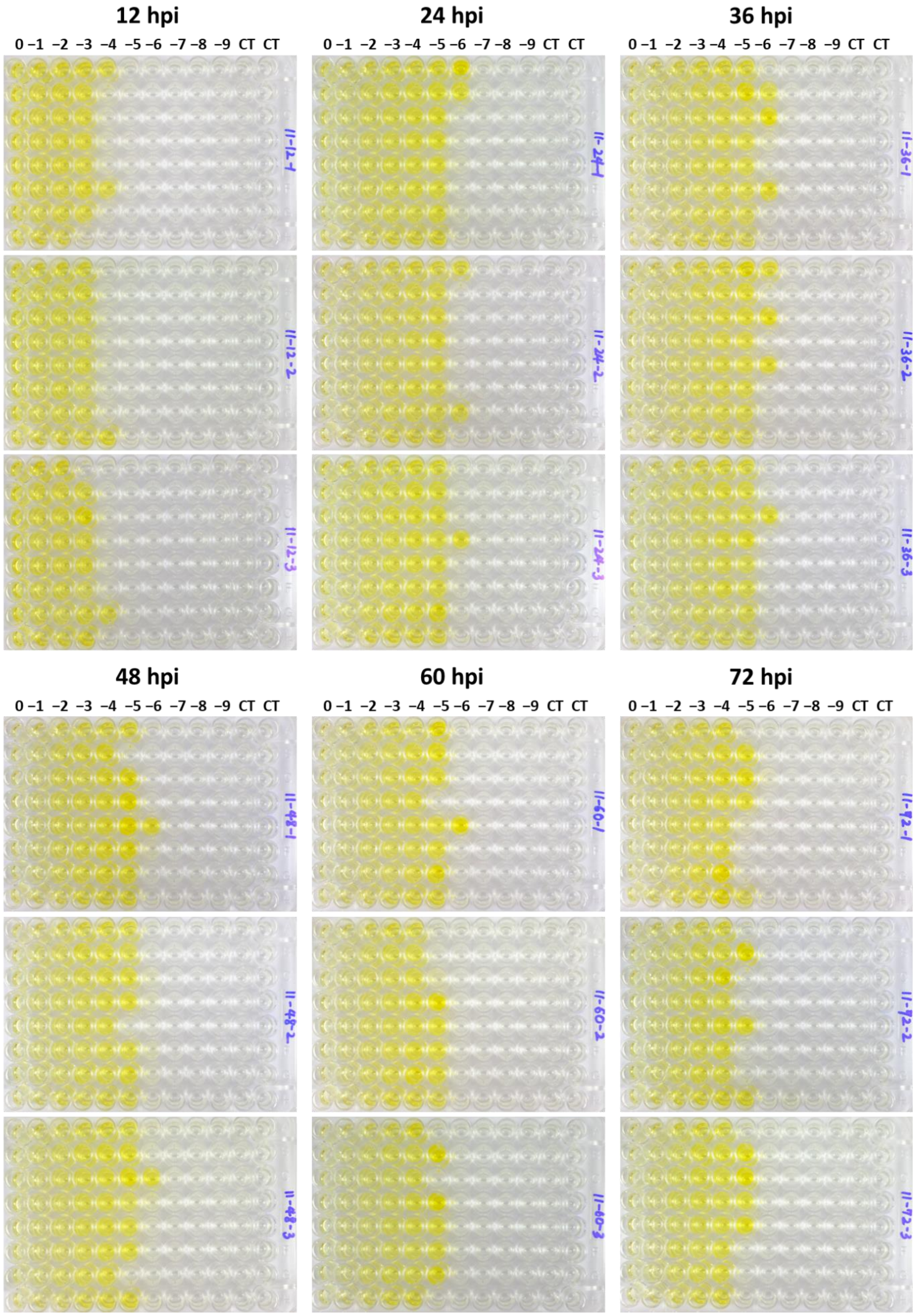

B

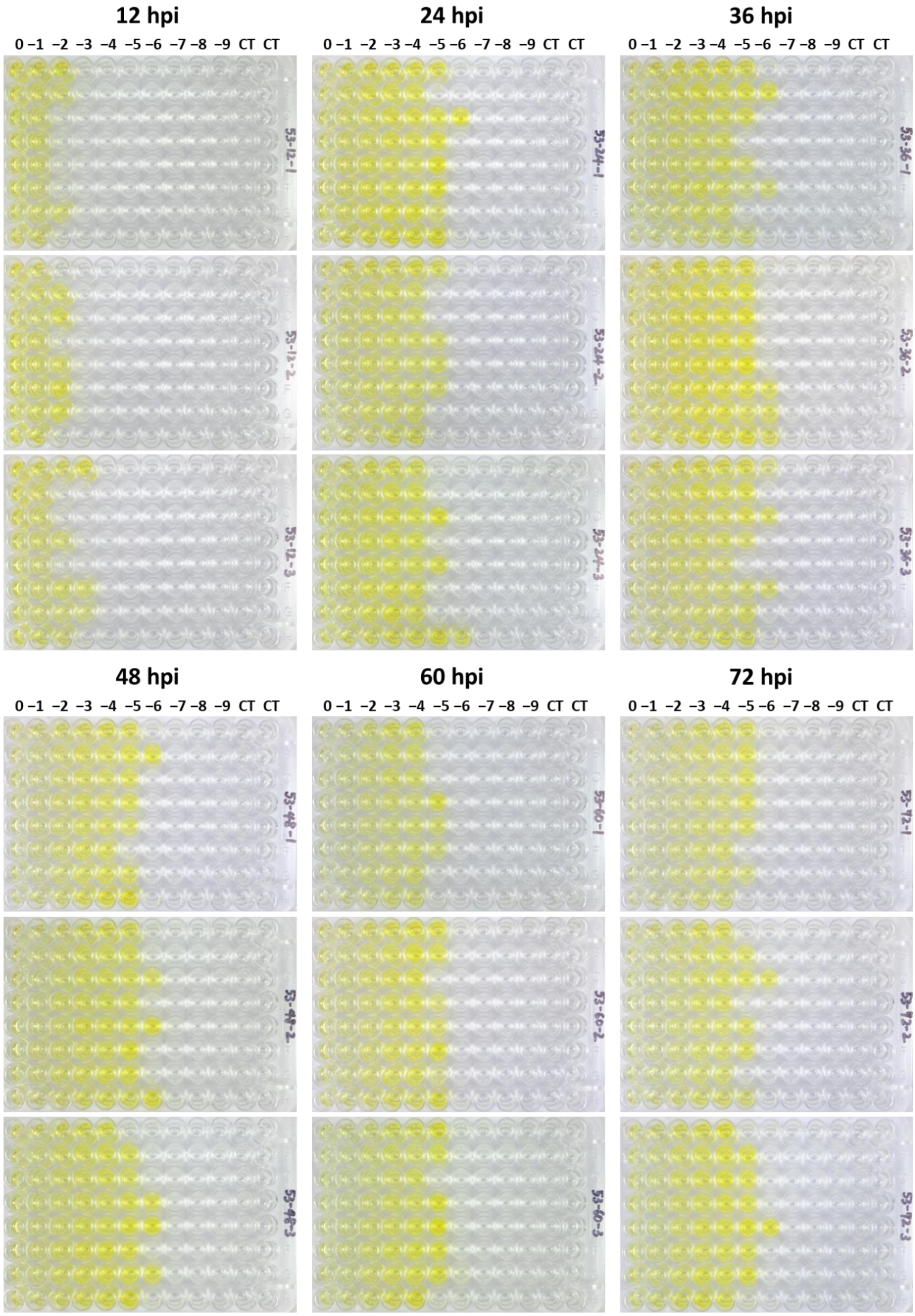

C

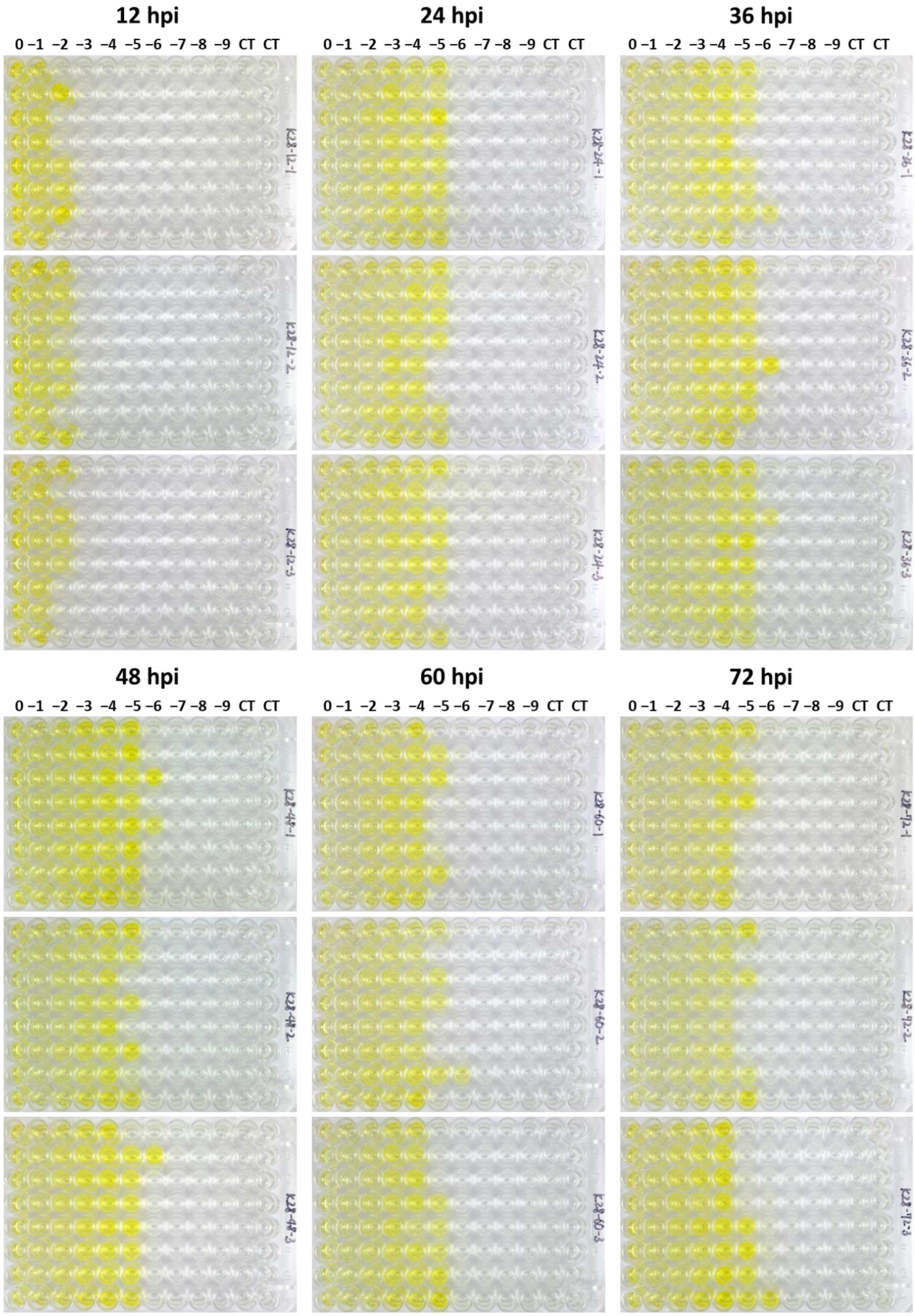

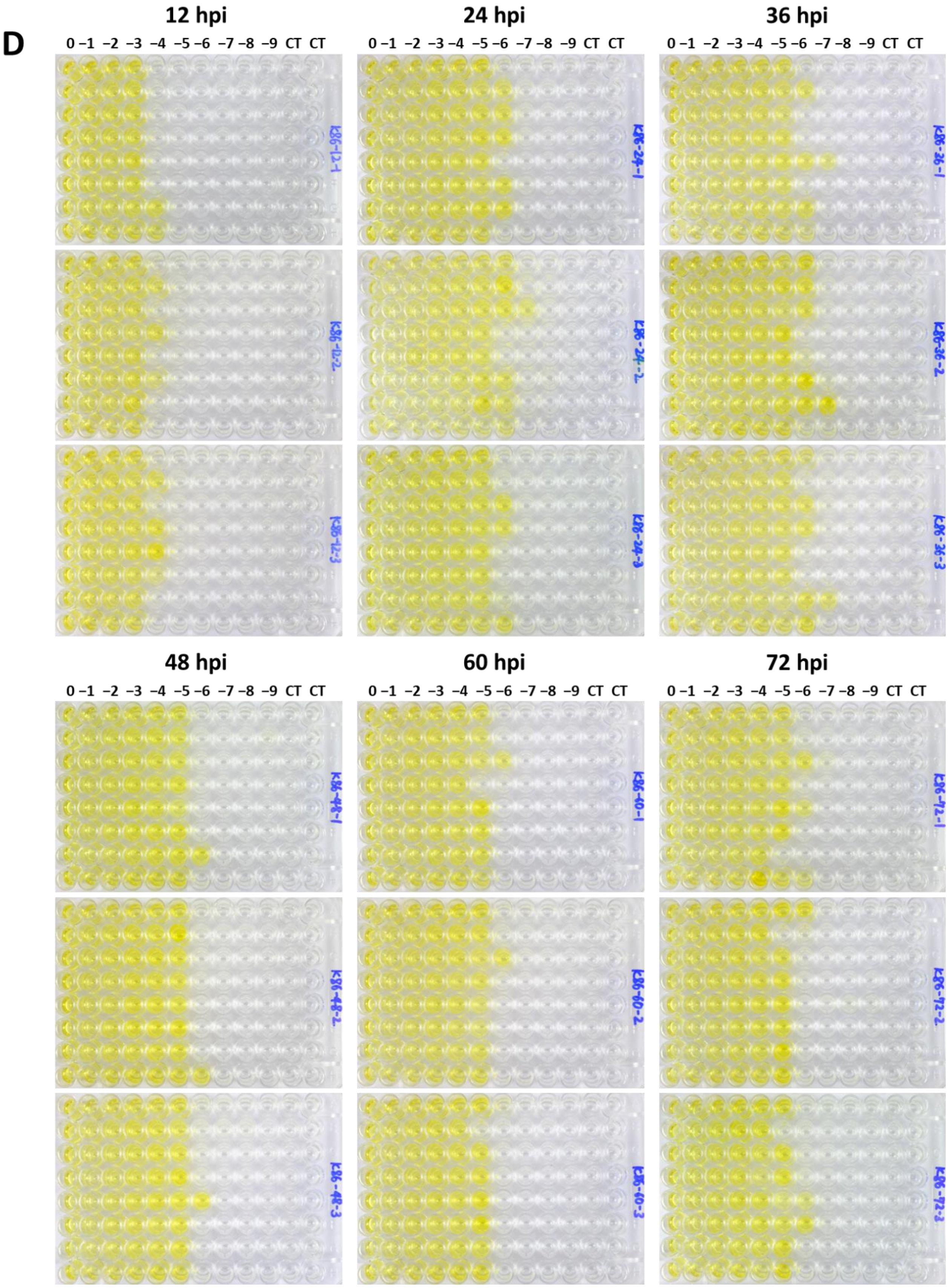
D
12 hpi
24 hpi
36 hpi
0 −1 −2 −3 −4 −5 −6 −7 −8 −9 CT CT
48 hpi
60 hpi
72 hpi
0 −1 −2 −3 −4 −5 −6 −7 −8 −9 CT CT

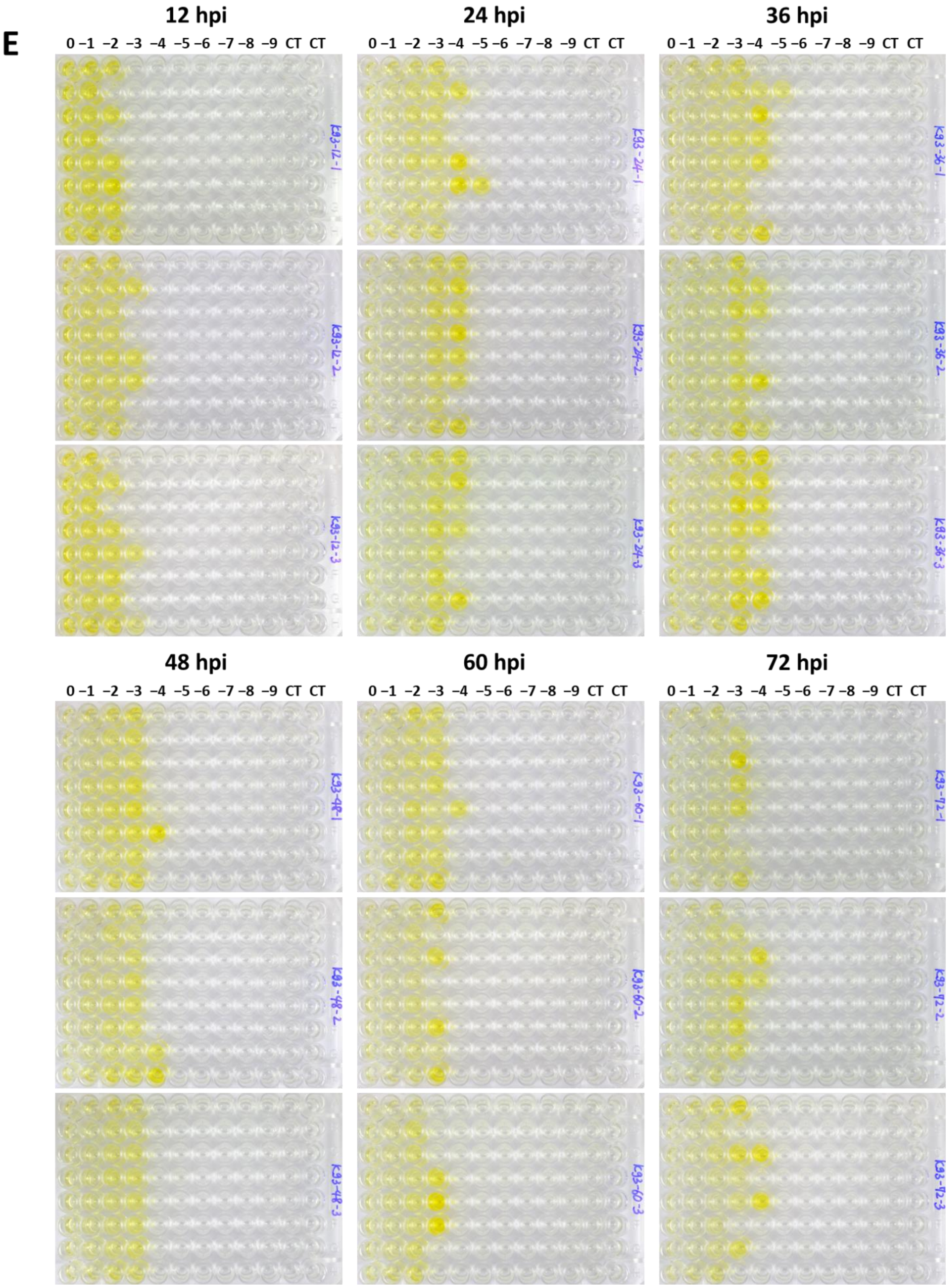

Figure S2. Raw ELISA data of $TCID_{50}$ assay to determine the viral growth kinetics in MDCK cells. (**A**) H1N1, (**B**) H5N3, (**C**) KNU18-28, (**D**) KNU18-86, and (**E**) KNU18-93. hpi: hour post infection.

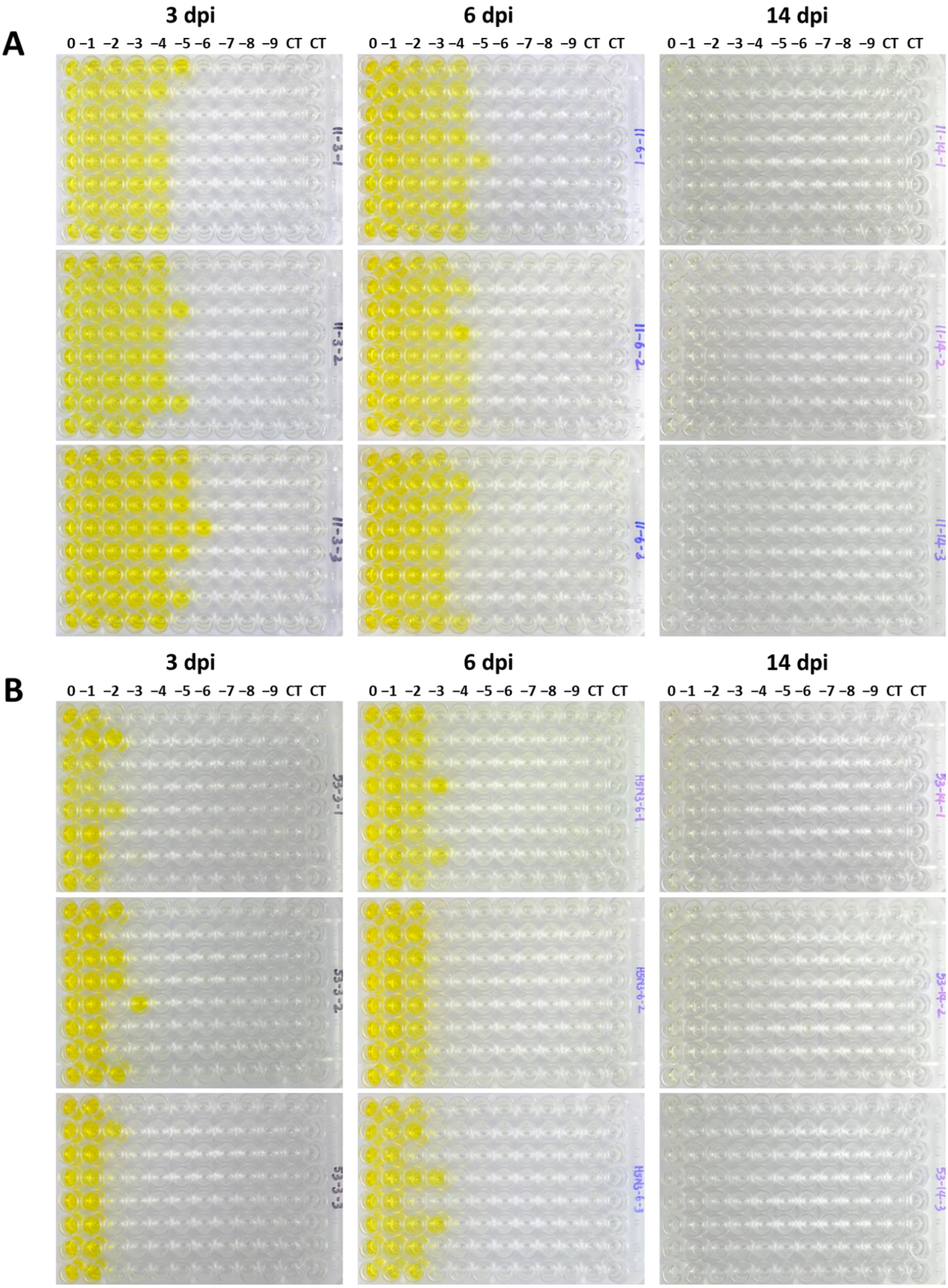
A
3 dpi
6 dpi
14 dpi
0 −1 −2 −3 −4 −5 −6 −7 −8 −9 CT CT
B
3 dpi
6 dpi
14 dpi
0 −1 −2 −3 −4 −5 −6 −7 −8 −9 CT CT

C

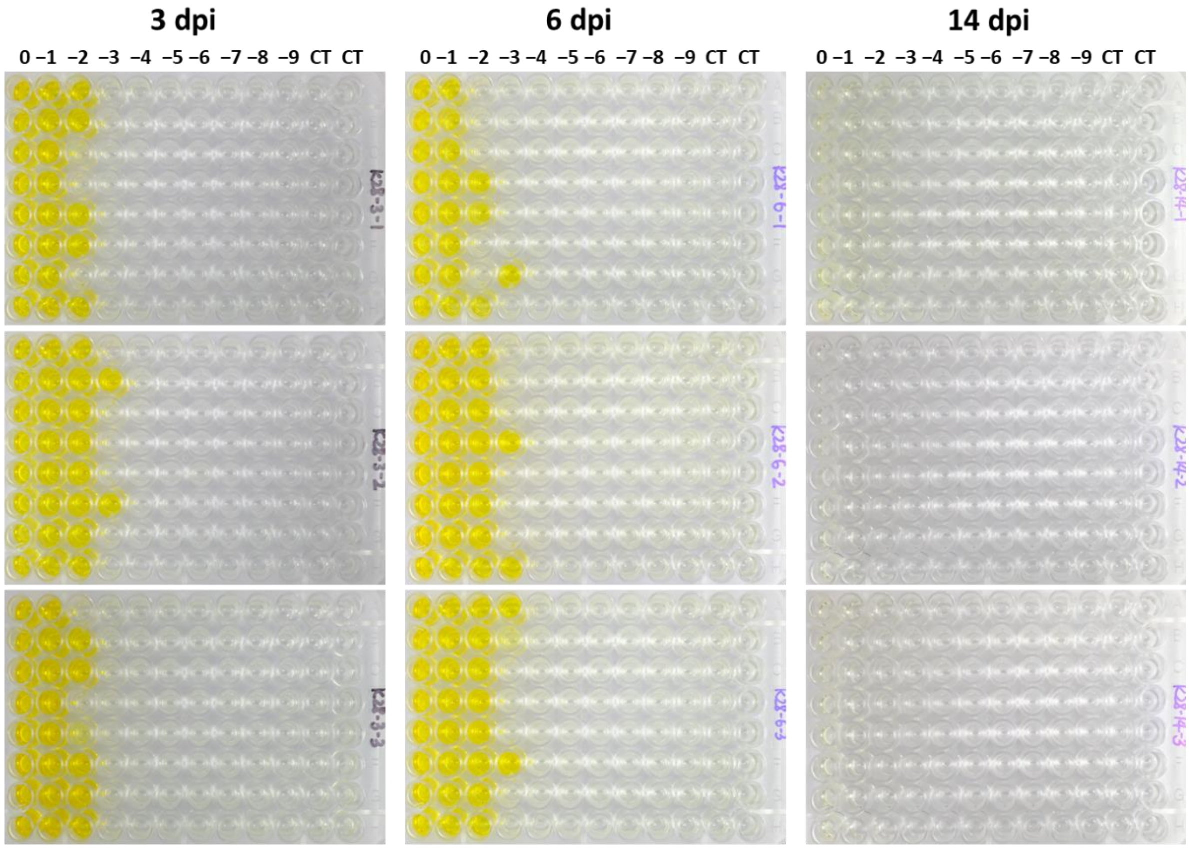
3 dpi
6 dpi
14 dpi
0 −1 −2 −3 −4 −5 −6 −7 −8 −9 CT CT
0 −1 −2 −3 −4 −5 −6 −7 −8 −9 CT CT
0 −1 −2 −3 −4 −5 −6 −7 −8 −9 CT CT

D

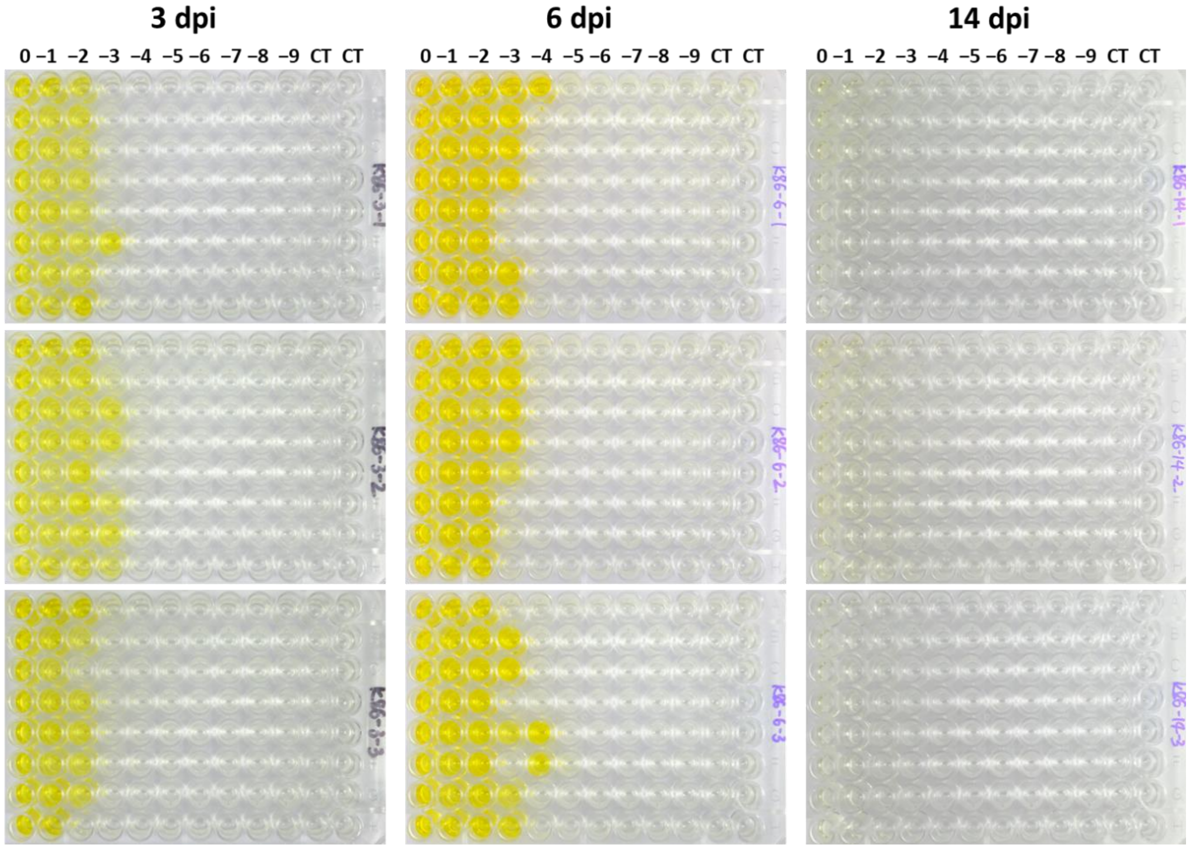
3 dpi
6 dpi
14 dpi
0 −1 −2 −3 −4 −5 −6 −7 −8 −9 CT CT
0 −1 −2 −3 −4 −5 −6 −7 −8 −9 CT CT
0 −1 −2 −3 −4 −5 −6 −7 −8 −9 CT CT

E

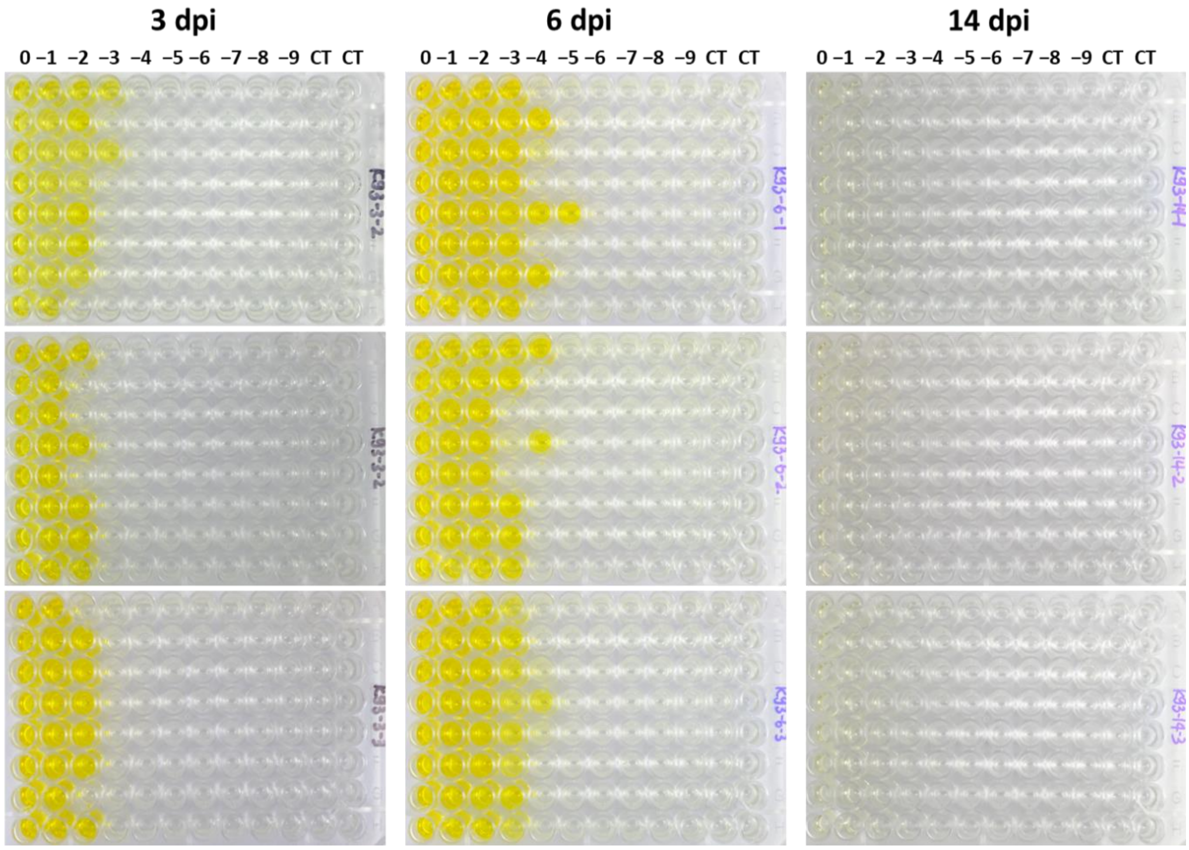

Figure S3. Raw ELISA data of $TCID_{50}$ assay for viral load shedding in infected mouse lung. (**A**) H1N1, (**B**) H5N3, (**C**) KNU18-28, (**D**) KNU18-86, and (**E**) KNU18-93. dpi: day post infection.

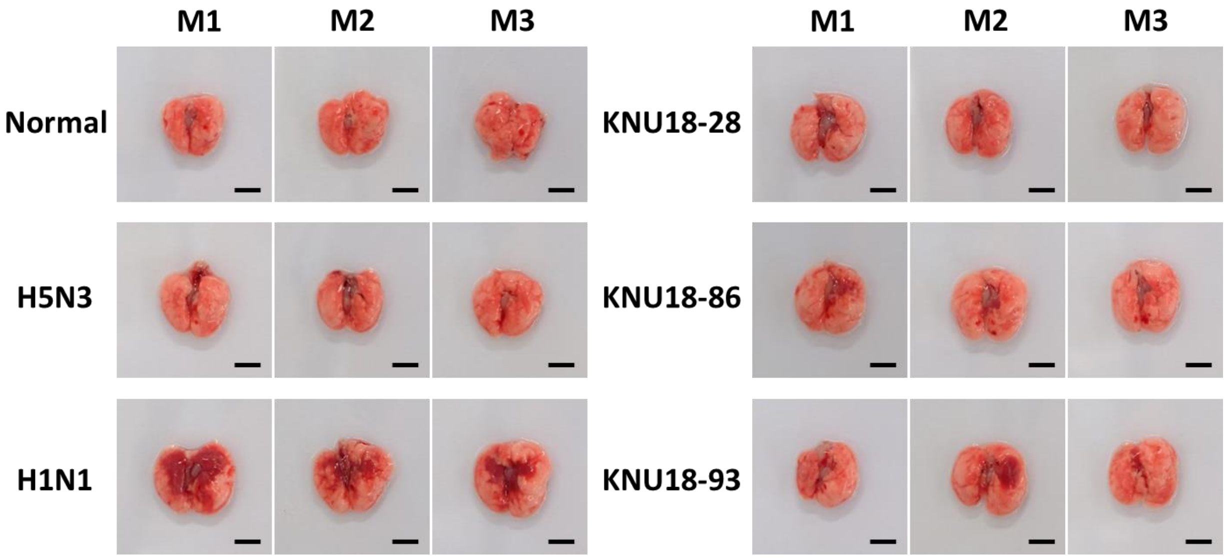

Figure S4. Lungs from mouse infected H1N1, H5N3, KNU18-28, KNU18-86, and KNU18-93 at 6 days post-infection. Scale bar 0.5 cm.

Table S1. Genome identity comparison of three novel H5N2 (KNU18-28, KNU18-86, and KNU18-93) isolates and H5N3 (KNU18-91) isolate.

Isolates	KNU18-91 (PB2)	KNU18-91 (PB1)	KNU18-91 (PA)	KNU18-91 (HA)	KNU18-91 (NP)	KNU18-91 (NA)	KNU18-91 (M)	KNU18-91 (NS)
**KNU18-28**	95.04%	93.98%	93.77%	98.53%	98.46%	-	98.68%	72.84%
**KNU18-86**	95.09%	94.02%	93.64%	98.47%	98.40%	-	98.68%	72.95%
**KNU18-93**	95.04%	93.93%	93.82%	98.47%	98.26%	-	98.68%	73.49%
